# S-GRAS score for prognostic classification of adrenocortical carcinoma: an international, multicenter ENSAT study

**DOI:** 10.1530/EJE-21-0510

**Published:** 2021-10-27

**Authors:** Y S Elhassan, B Altieri, S Berhane, D Cosentini, A Calabrese, M Haissaguerre, D Kastelan, M C B V Fragoso, J Bertherat, A Al Ghuzlan, H Haak, M Boudina, L Canu, P Loli, M Sherlock, O Kimpel, M Laganà, A J Sitch, M Kroiss, W Arlt, M Terzolo, A Berruti, J J Deeks, R Libé, M Fassnacht, C L Ronchi, the ENSAT

**Affiliations:** 1 Institute of Metabolism and Systems Research, University of Birmingham, Birmingham, UK; 2 Department of Endocrinology, Queen Elizabeth Hospital, University Hospitals Birmingham NHS Foundation Trust, Birmingham, UK; 3 Division of Endocrinology and Diabetes, Department of Internal Medicine I, University Hospital, University of Würzburg, Würzburg, Germany; 4 NIHR Birmingham Biomedical Research Centre, University Hospitals Birmingham NHS Foundation Trust and University of Birmingham, Birmingham, UK; 5 Institute of Applied Health Research, University of Birmingham, Birmingham, UK; 6 Medical Oncology, Department of Medical and Surgical Specialties, Radiological Sciences and Public Health University of Brescia, ASST-Spedali Civili, Brescia, Italy; 7 Department of Clinical and Biological Sciences, University of Turin, San Luigi Hospital, Orbassano, Italy; 8 Service d’Endocrinologie – Diabète et Nutrition CHU de Bordeaux, Bordeaux, France; 9 Department of Endocrinology, University Hospital Centre Zagreb, Zagreb, Croatia; 10 Unidade de Suprarrenal da Disciplina de Endocrinologia e Metabologia da Faculdade de Medicina do Hospital das Clinicas da Universidade de São Paulo (HCFMUSP), and Instituto do Cancer do Estado de Sao Paulo (ICESP), Sao Paulo, Brazil; 11 Reference Center for Rare Adrenal Cancer (COMETE), Cochin Hospital, Paris, France; 12 Department of Pathology, Gustave Roussy Cancer Center, Paris, France; 13 Department of Internal Medicine, Máxima MC, Eindhoven, Netherlands; 14 Department of Endocrinology, Theagenio Cancer Hospital, Thessaloniki, Greece; 15 Department of Experimental and Clinical Biomedical Sciences, University of Florence, Florence, Italy; 16 Clinica Polispecialistica San Carlo, Paderno Dugnano, Milano, Italy; 17 Department of Endocrinology, Beaumont Hospital, and the Royal College of Surgeons, Dublin, Republic of Ireland; 18 Comprehensive Cancer Center Mainfranken, University of Würzburg, Würzburg, Germany; 19 Department for Endocrinology, Medizinische Klinik und Poliklinik IV, Ludwig-Maximilians-University, Munich, Germany; 20 Department of Endocrinology and Metabolic Diseases, Hôpital Cochin, Paris, France

## Abstract

**Objective:**

Adrenocortical carcinoma (ACC) has an aggressive but variable clinical course. Prognostic stratification based on the European Network for the Study of Adrenal Tumours stage and Ki67 index is limited. We aimed to demonstrate the prognostic role of a points-based score (S-GRAS) in a large cohort of patients with ACC.

**Design:**

This is a multicentre, retrospective study on ACC patients who underwent adrenalectomy.

**Methods:**

The S-GRAS score was calculated as a sum of the following points: tumour stage (1–2 = 0; 3 = 1; 4 = 2), grade (Ki67 index 0–9% = 0; 10–19% = 1; ≥20% = 2 points), resection status (R0 = 0; RX = 1; R1 = 2; R2 = 3), age (<50 years = 0; ≥50 years = 1), symptoms (no = 0; yes = 1), and categorised, generating four groups (0–1, 2–3, 4–5, and 6–9). Endpoints were progression-free survival (PFS) and disease-specific survival (DSS). The discriminative performance of S-GRAS and its components was tested by Harrell’s Concordance index (C-index) and Royston–Sauerbrei’s R^2^_D_ statistic.

**Results:**

We included 942 ACC patients. The S-GRAS score showed superior prognostic performance for both PFS and DSS, with best discrimination obtained using the individual scores (0–9) (C-index = 0.73, R^2^_D_ = 0.30, and C-index = 0.79, R^2^_D_ = 0.45, respectively, all *P* < 0.01vs each component). The superiority of S-GRAS score remained when comparing patients treated or not with adjuvant mitotane (*n* = 481 vs 314). In particular, the risk of recurrence was significantly reduced as a result of adjuvant mitotane only in patients with S-GRAS 4–5.

**Conclusion:**

The prognostic performance of S-GRAS is superior to tumour stage and Ki67 in operated ACC patients, independently from adjuvant mitotane. S-GRAS score provides a new important guide for personalised management of ACC (i.e. radiological surveillance and adjuvant treatment).

## Introduction

Adrenocortical carcinoma (ACC) is a rare malignancy with an incidence of 0.7–2/million/year (1, 2). Although prognosis in ACC is generally unfavourable, there is wide heterogeneity in the outcomes. The 5-year survival ranges from 10 to 60%, mostly reflecting the primary tumour stage and the resection status (3, 4). However, up to 60% of patients with localised tumours experience disease recurrence after radical surgery (5) and 15% die within 2 years (3). Reliable prognostication after ACC resection is critical to guide frequency of follow-up, adjuvant treatment, and to more accurately counsel patients regarding long-term outcomes.

Nonetheless, the optimal tools for prognostication in ACC are debatable. Despite the considerable heterogeneity, the European Network for the Study of Adrenal Tumours (ENSAT) staging system is widely used as the standard prognostic factor in ACC (3, 6, 7). To better prognosticate patients with advanced disease, a modified ENSAT stage has been proposed which sub-classifies patients to account for involved regional lymph nodes and number of metastatic organs (mENSAT stages 4a, 4b, and 4c) (8). Among other prognostic characteristics, the Ki67 proliferation index (9) is considered the most important single parameter (6, 10, 11). Incomplete tumour resection is also linked to unfavourable prognosis (12, 13). Finally, although older age and steroid excess have been associated with decreased survival (14, 15, 16), their prognostic value remains uncertain (17, 18).

Due to the shortcomings of individual clinical/histopathological prognostic markers, combined scores were studied for better prognostic accuracy in ACC. We firstly demonstrated the prognostic value of the GRAS components, that is, grading (G, Weiss score >6 and/or Ki67 ≥20%), resection status (R), age (A) and tumour- or hormone-related symptoms (S)) in 444 patients with advanced ACC (8). More recently, in 107 patients, we reported that a modified form of the GRAS classification, here termed S-GRAS, which includes the ENSAT stage and focuses on Ki67 for grading, allows better stratification than individual clinical/histopathological characteristics (19).

Herein, we aimed to demonstrate the prognostic performance of the S-GRAS score in the largest study cohort of well-characterised ACC patients to-date. We also investigated the S-GRAS prognostic role in relation to adjuvant mitotane treatment, being the most utilised adjuvant therapy in ACC (20, 21).

## Methods

### Patients and data collection

This is a retrospective, international, multicentre study conducted on behalf of ENSAT (www.ensat.org). Details of the recruitment process and data collection are reported in Supplementary Table 1 (see section on supplementary materials given at the end of this article). The inclusion criteria were: (i) age ≥ 18 years, (ii) histologically confirmed ACC, (iii) available clinical/histopathological characteristics at diagnosis to allow the calculation of the S-GRAS score, and (iv) available follow-up radiological data to determine disease status and survival. We excluded non-operated ACC patients and those included in our two previous studies (Libé *et al*. (8) and Lippert *et al.* (19)). Given the exclusion of patients with inoperable disease, we did not consider the previously proposed mENSAT classification for patients with advanced ACC (8).

Mandatory data included: sex, age at diagnosis, presence of tumour- or hormone-related symptoms at presentation, adrenal hormone status, ENSAT stage, date of adrenalectomy, resection status (R0 = complete resection, RX = cannot be assessed, R1 = indicates the removal of all macroscopic disease, but microscopic margins are positive for tumour, or R2 = indicates gross residual disease with post-operative residual disease that was not resected (primary tumour, regional nodes, and macroscopic margin involvement), Ki67 index, date of disease recurrence or progression after primary surgery, and date of last follow-up or death. Symptoms were defined as: hormone-related if due to adrenal hormone excess (e.g. glucocorticoids – Cushing syndrome; androgens – hirsutism, acne, and alopecia; mineralocorticoids – uncontrolled blood hypertension), tumour-related if due to mass effect (e.g. abdominal pain), or systemic cancer-related (e.g. fatigue or weight loss). Patients with ENSAT stage 4 with complete resection of primary tumours and metastases were considered R0. Patients with ENSAT stage 4 with residual disease manifestations were defined as R2.

We calculated the S-GRAS score as previously described (19): age at diagnosis (<50 years = 0 point; ≥50 years = 1 point), hormone, tumour or systemic cancer-related symptoms at presentation (no = 0 point; yes = 1 point), ENSAT stage (1 or 2 = 0 point; 3 = 1 point; 4 = 2 points), R of primary tumour (R0 = 0 point; RX = 1 point; R1 = 2 points; R2 = 3 points), and Ki67 index (0–9% = 0 point; 10–19% = 1 point; ≥20% = 2 points) (Supplementary Table 2), generating ten S-GRAS scores and four S-GRAS groups: 0–1, 2–3, 4–5, and 6–9 (19). Given the exclusion of patients with inoperable disease, we did not consider the previously proposed mENSAT classification for patients with advanced ACC (8).

Disease monitoring was done through periodical cross-sectional imaging as detailed in Supplementary Table 1. We also collected details of whether adjuvant treatment with mitotane (o,p'-DDD) was used after radical surgery or not (21). Modalities of initiation and titration of mitotane treatment are reported in Supplementary Table 1.

All participating institutions obtained local ethics approval for recording of pseudonymised and standardised data in the ENSAT registry for use in any current and future adrenal tumour-related projects (www.ensat.org). The entire list of the participating centres as well as the details of the ethical approval are provided in the Supplementary Table 1. All participants provided written informed consent.

### Endpoints

Primary outcomes were: (i) progression-free survival (PFS) defined as the time from primary tumour resection to the first radiological evidence of progression (e.g. disease relapse in patients after radical resection or progressive and/or new lesions in patients with advanced disease, i.e. R2, as defined by local radiologists) and (ii) disease-specific survival (DSS) defined as the time from primary resection of ACC to disease-related death.

DSS was chosen against overall survival to have a more accurate measure of the clinical prognosis, avoiding a potential overestimation of mortality (especially of older patients).

### Statistical analysis

Statistical analysis was undertaken using Stata/SE Version 16.0 (StataCorp).

*Post hoc* power calculation was based on the method described by Jinks *et al*. (22) (Stata command *dsampsi*) that relies on a target value of the Royston and Sauerbrei D measure of discrimination (22). Details are reported in the Supplementary Table 3. With 942 patients in this study, we have adequate sample size for the evaluation of S-GRAS to cover a range of possible values of D.

Continuous variables were presented as median and interquartile range and categorical variables as counts and percentages. For each of the DSS and PFS endpoints, we performed Kaplan–Meier (KM) survival curves according to the S-GRAS score, ENSAT stage, Ki67 index, resection status, age, and symptoms. Median survival, percentage survival at 24 and 60 months were reported for each survival curve. The prognostic effect of S-GRAS score and its individual components was examined using univariable Cox regression. Hazard ratio (HR), 95% CIs, and *P*-values were reported. *P*-values <0.05 were considered significant. The discriminative performance of the four S-GRAS groups and ten individual scores were compared to the single components using the Harrell’s Concordance index (C-index) (23), and the Royston–Sauerbrei’s Discrimination R^2^_D_ statistic (24). Harrell’s C-index is the proportion of patient pairs where the predicted and observed survival outcomes are in agreement with respect to rank. R^2^_D_ measures the level of explained variation on the log relative hazard scale. A higher value of Harrell’s C-index and R^2^_D_ is indicative of better model discrimination.

Finally, the added value of resection, age, and symptom status (RAS components) was evaluated by comparing multivariable Cox regression models with and without these three factors added to ENSAT tumour stage (S) and Ki67 index (G) using a likelihood ratio test.

#### Subgroup analyses

The performance of S-GRAS score and its components was also assessed within patients with R0 resection status for the recurrence-free survival (RFS), defined as the time from primary adrenalectomy to the first radiological evidence of relapse (*n* = 648).

Excluding patients with R2 status and those with incomplete treatment data, a subgroup analysis was performed comparing patients treated with adjuvant mitotane (*n* = 481) with those untreated (*n* = 314). Aforementioned methods were repeated, but an interaction term was introduced to the univariable Cox regressions to determine if the prognostic effect of S-GRAS score, ENSAT stage, Ki67 index, and resection status varied according to mitotane treatment status. Specifically, marginal effect statistics were derived from univariable Cox regressions that included an interaction term between each score and mitotane treatment status. These measured the change in predicted HR if status changed from no treatment to treatment. KM survival curves according to treatment status were plotted. The C-index and R^2^_D_ statistic were also reported for each subgroup.

## Results

### Patient demographics

The 14 participating ENSAT centres identified 1075 ACC patients who underwent adrenalectomy between 2010 and 2019 (Supplementary Fig. 1). A final cohort of 942 eligible patients were enrolled. The baseline characteristics and follow-up data are summarised in Supplementary Table 4. Briefly, 62% of patients were women, 70% presented with symptoms, 50% had ENSAT stage 2 disease, 69% had R0 status, 49% had Ki67 index ≥20%, and most developed disease recurrence or progression during follow-up (61%). Overall, 281 patients died of ACC (30%), while additional 14 patients died for other reasons (2%, 9 of whom were tumour-free at the time of death).

### Prognostic performance of the four S-GRAS score groups

#### Progression-free survival

**Figure 1:**
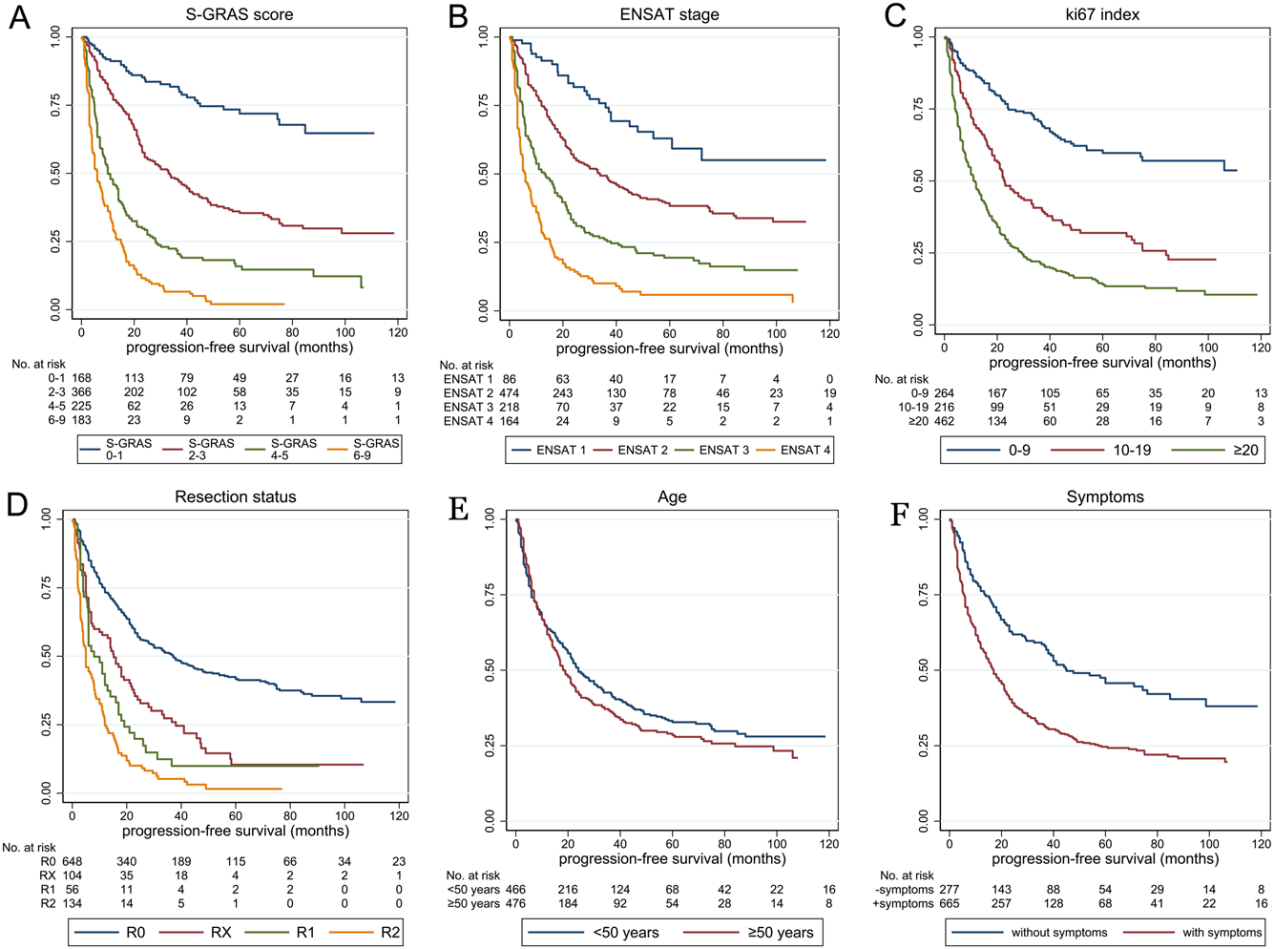
Kaplan–Meier curves depicting progression-free survival according to S-GRAS score grouping and each S-GRAS component (*n* = 942). (A) S-GRAS groups 0–1, 2–3, 4–5, and 6–9, (B) ENSAT tumour stage (1–4), (C) Ki67 proliferation index (0–9, 10–19, ≥20), (D) resection status of primary tumour (R0, RX, R1, and R2), (E) age at time of diagnosis (<50, ≥50), (F) symptoms at time of diagnosis. For univariate statistical analysis, see Table 1.

KM curves for the S-GRAS groups and its components are shown in Fig. 1A, B, C, D, E and  F. Univariable survival analysis showed all the evaluated variables being strongly associated with PFS, except age (Table 1). The discrimination statistics showed the superiority of S-GRAS compared to all its components (Harrell’s C-index *P* < 0.0001vseach component, *P* = 0.003 vs continuous Ki67, Table 1). Specifically, the S-GRAS groups showed better performance (C-index = 0.71, R^2^_D_ = 0.30) compared to ENSAT staging (C-index = 0.67, R^2^_D_ = 0.21), and Ki67 index (C-index = 0.65, R^2^_D_ = 0.21 for 0–9%, 10–19%, and ≥20% grouping). There was a significantly higher risk of disease progression in patients with S-GRAS 2–3, 4–5, and 6–9 compared to S-GRAS 0–1 (2.8, 6.4, and 11.5 times, respectively, *P* < 0.0001 for all, Table 1). Moreover, S-GRAS 0–1 identified a larger group of ACC patients with longer PFS (*n* = 168, 72% PFS at 60 months) compared to ENSAT stage 1 (*n* = 86, 63% PFS at 60 months).

**Table 1:** Univariate survival analysis: median progression-free survival, percentage survival at 24 and 60 months, hazard ratios and discrimination analysis of single prognostic variables and S-GRAS scoring (*n* = 942).

Variables	*n*	% survival		Median PFS, months (95% CI)	HR (95% CI)	*P*-value (HR)	Harrell’s C-index (95% CI)	*P*-values (Harrell’s C-index)	Royston–Sauerbrei’s R^2^_D_ statistic
		At 24 months	At 60 months						
ENSAT stage							0.67 (0.65, 0.69)	<0.0001*; <0.0001**	0.21 (0.16, 0.26)
1	86	81.7 (71.0, 88.8)	63.0 (49.7, 73.6)	NR	1	Reference			
2	474	56.0 (51.1, 60.7)	38.3 (33.0, 43.6)	34.0 (25.0, 42.4)	2.1 (1.4, 3.1)	<0.0001			
3	218	32.1 (25.6, 38.8)	19.4 (13.7, 25.8)	13.5 (9.3, 17.0)	4.1 (2.7, 6.2)	<0.0001			
4	164	15.0 (9.7, 21.5)	5.9 (2.5, 11.3)	6.6 (4.2, 8.0)	7.7 (5.1, 11.8)	<0.0001			
Ki67 index							0.65 (0.63, 0.67)	<0.0001*; <0.0001**	0.21 (0.16, 0.27)
0–9	264	75.2 (69.0, 80.3)	59.7 (52.1, 66.5)	127.0 (75.0, NR)	1	Reference			
10–19	216	48.3 (41.0, 55.3)	31.9 (24.7, 39.3)	22.8 (19.0, 33.0)	2.3 (1.8, 3.0)	<0.0001			
≥20	462	28.5 (24.2, 33.0)	13.9 (10.3, 18.1)	11.4 (9.8, 13.6)	4.1 (3.2, 5.2)	<0.0001			
Ki67 (continuous)	942	–	–	–	1.03 (1.02, 1.03)	<0.0001	0.68 (0.65, 0.70)	0.003*, <0.0001**	0.16 (0.12, 0.20)
Resection status							0.64 (0.62, 0.66)	<0.0001*; <0.0001**	0.24 (0.19, 0.29)
R0	648	57.2 (53.0, 61.2)	41.7 (37.1, 46.1)	37.1 (29.4, 45.0)	1	Reference			
RX	104	34.1 (24.5, 43.9)	10.4 (4.3, 19.6)	15.2 (8.0, 21.0)	2.0 (1.6, 2.6)	<0.0001			
R1	56	19.8 (10.0, 32.2)	9.9 (3.3, 20.9)	8.0 (6.0, 14.0)	2.8 (2.1, 3.9)	<0.0001			
R2	134	10.0 (5.4, 16.4)	1.6 (0.2, 6.6)	5.0 (4.0, 7.6)	4.6 (3.7, 5.7)	<0.0001			
Age							0.51 (0.49, 0.53)	<0.0001*; <0.0001**	0.004 (0.000, 0.020)
<50 years	466	49.5 (44.6, 54.2)	32.8 (27.8, 37.8)	24.0 (21.0, 31.0)	1	Reference			
≥50 years	476	42.3 (37.5, 47.1)	28.5 (23.7, 33.4)	18.2 (16.3, 21.6)	1.1 (1.0, 1.3)	0.140			
Age (continuous)	942	–	–	–	1.01 (1.00, 1.01)	0.033	0.52 (0.49, 0.54)	<0.0001*, <0.0001**	0.01 (0.00, 0.02)
Symptoms							0.57 (0.55, 0.59)	<0.0001*; <0.0001**	0.08 (0.04, 0.14)
No	277	62.4 (55.8, 68.2)	45.7 (38.4, 52.7)	45.0 (37.2, 76.1)	1	Reference			
Yes	665	39.2 (35.3, 43.2)	24.6 (20.9, 28.6)	16.9 (14.3, 20.0)	1.9 (1.6, 2.3)	<0.0001			
S-GRAS group							0.71 (0.69, 0.73)	Reference 1*	0.30 (0.25, 0.35)
0-1	168	84.4 (77.4, 89.4)	71.9 (62.6, 79.3)	181.6 (127.0, NR)	1	Reference			
2-3	366	55.2 (50.1, 61.1)	35.4 (29.5, 41.4)	33.0 (25.0, 40.8)	2.8 (2.0, 4.0)	<0.0001			
4-5	225	29.2 (23.1, 35.6)	15.9 (10.6, 22.2)	10.0 (8.2, 14.0)	6.4 (4.5, 9.0)	<0.0001			
6-9	183	11.5 (7.1, 17.1)	2.0 (0.4, 6.0)	6.0 (4.3, 8.0)	11.5 (8.1, 16.3)	<0.0001			
S-GRAS individual score							0.73 (0.71, 0.75)	Reference 2**	0.30 (0.25, 0.34)
0	36	93.6 (76.5, 98.4)	74.7 (51.2, 88.0)	NR	0.7 (0.3, 1.6)	0.373			
1	132	81.9 (73.5, 87.8)	71.2 (60.7, 79.5)	181.6 (127.0, NR)	1	Reference			
2	155	69.9 (61.3, 76.9)	45.1 (34.9, 54.8)	48.0 (37.2, NR)	1.9 (1.2, 2.9)	0.003			
3	211	46.0 (38.6, 53.0)	28.6 (21.7, 35.8)	23.0 (21.0, 29.0)	3.3 (2.2, 4.8)	<0.0001			
4	153	31.7 (24.1, 39.6)	20.6 (13.6, 28.6)	13.5 (10.0, 16.0)	5.1 (3.5, 7.5)	<0.0001			
5	72	24.1 (14.5, 35.1)	5.9 (1.3, 16.1)	6.0 (5.0, 11.0)	8.7 (5.7, 13.3)	<0.0001			
6	32	27.4 (12.0, 45.4)	9.1 (1.6, 24.9)	6.0 (4.0, 21.0)	6.9 (4.11, 11.6)	<0.0001			
7	39	7.9 (2.0, 19.1)	–	8.0 (4.0, 11.8)	10.2 (6.4, 16.5)	<0.0001			
8	75	11.4 (5.3, 20.0)	–	4.3 (3.5, 7.2)	12.8 (8.5, 19.57)	<0.0001			
9	37	–	–	5.0 (3.0, 12.0)	13.7 (8.3, 22.6)	<0.0001			

NR, not reached (= median survival not reached, i.e. percentage survival remained >50% so this value and/or its 95% CIs cannot be computed).

*P-values comparing the variable with the S-GRAS group. **P-values comparing the variable with the S-GRAS scores.

Finally, the comparison between the multivariable Cox regression models with and without resection, age, and symptom status added to ENSAT stage and Ki67 index showed that adding the three variables resulted in a significant improvement (*P* < 0.0001) in the model fit (Supplementary Table 5A). The Harrell’s C-index was also higher in the larger model (0.733 vs 0.717).

#### Disease-specific survival

**Figure 2:**
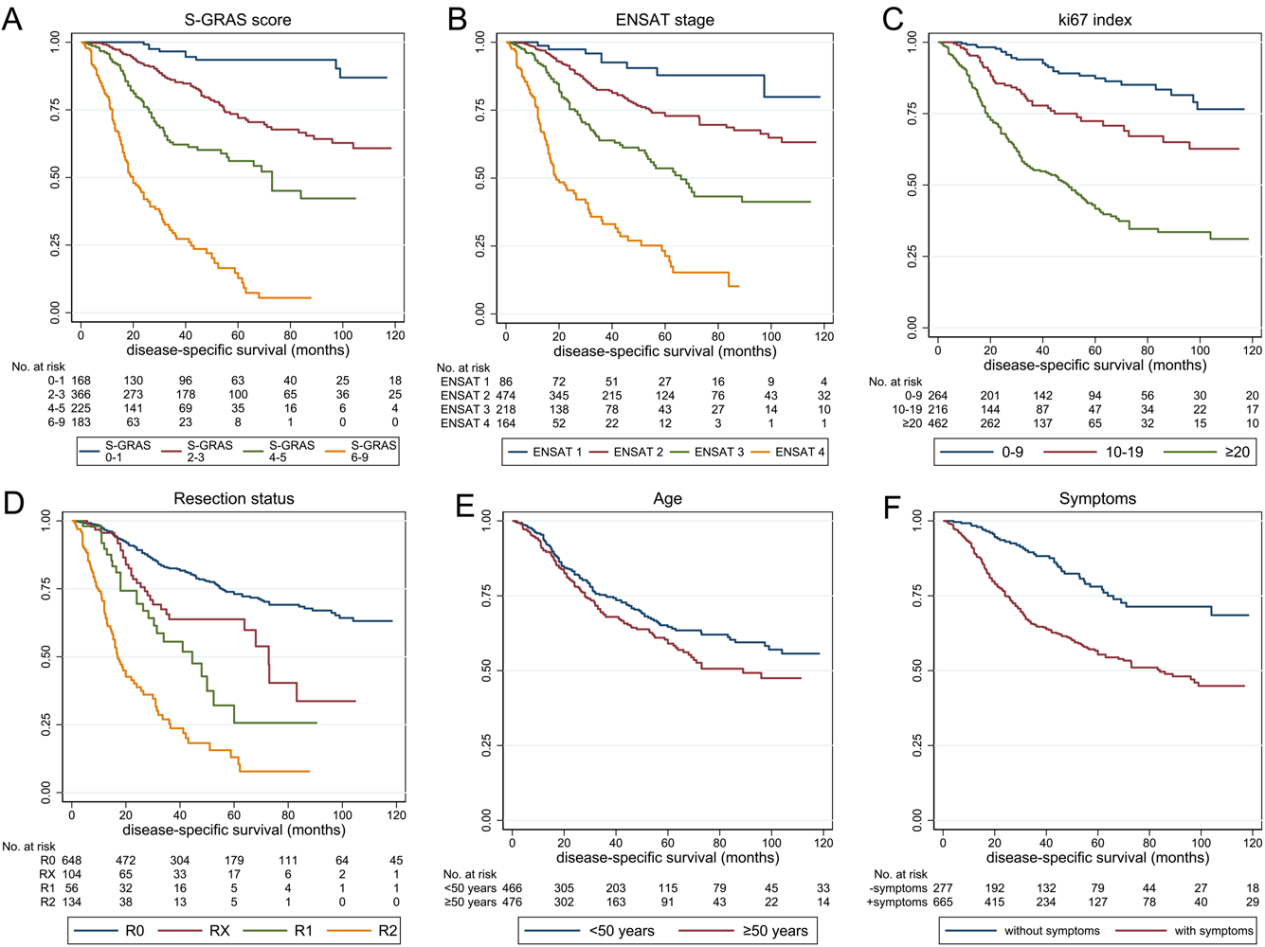
Kaplan–Meier curves depicting disease-specific survival according to S-GRAS score grouping and each S-GRAS component (*n* = 942). (A) S-GRAS groups 0–1, 2–3, 4–5, and 6–9, (B) ENSAT tumour stage (1–4), (C) Ki67 proliferation index (0–9, 10–19, ≥20), (D) resection status of primary tumour (R0, RX, R1, and R2), (E) age at time of diagnosis (<50, ≥50), (F) symptoms at time of diagnosis. For univariate statistical analysis, see Table 1.

As the number of ACC-unrelated deaths was small (14 out of 295 total deaths), there was no statistically significant difference between overall survival and DSS (median 104 vs 123 months, *P* = 0.4764). Therefore, DSS was considered as an approximate of overall survival. Univariable analysis showed all variables being strongly associated with DSS (Supplementary Table 6). Again, the four S-GRAS groups performed better compared to the individual components (*P* < 0.0001 for Harrell’s C-index), showing higher C-index and R^2^_D_ (C-index = 0.77, R^2^_D_ = 0.46) than ENSAT stage (C-index = 0.72, R^2^_D_ = 0.35) and Ki67 index (C-index = 0.69, R^2^_D_ = 0.31 for 0–9%, 10–19%, and ≥20% grouping). Compared to S-GRAS 0–1, patients with S-GRAS 2–3, 4–5, and 6–9 had 3.4, 7.8, and 27.3 times higher risk of disease-related death, respectively (Fig. 2A, B, C, D, E, and  F; *P < 0.0001*for all). S-GRAS group 0–1 patients had longer DSS (*n* = 168, 93% DSS at 60 months) compared to ENSAT stage 1 (*n* = 86, 88% DSS at 60 months, Supplementary Table 6), and S-GRAS group 6–9 showed worse clinical outcome than ENSAT4 (13 vs 21% DSS at 60 months).

As with the PFS outcome, multivariable Cox regression models showed that adding resection status, age, and symptoms to ENSAT stage and Ki67 resulted in a significant improvement in model fit (*P* < 0.0001) (Supplementary Table 5B). This led to an increase in the Harrell’s C-index from 0.771 to 0.797.

### Prognostic performance of the individual S-GRAS scores

**Figure 3:**
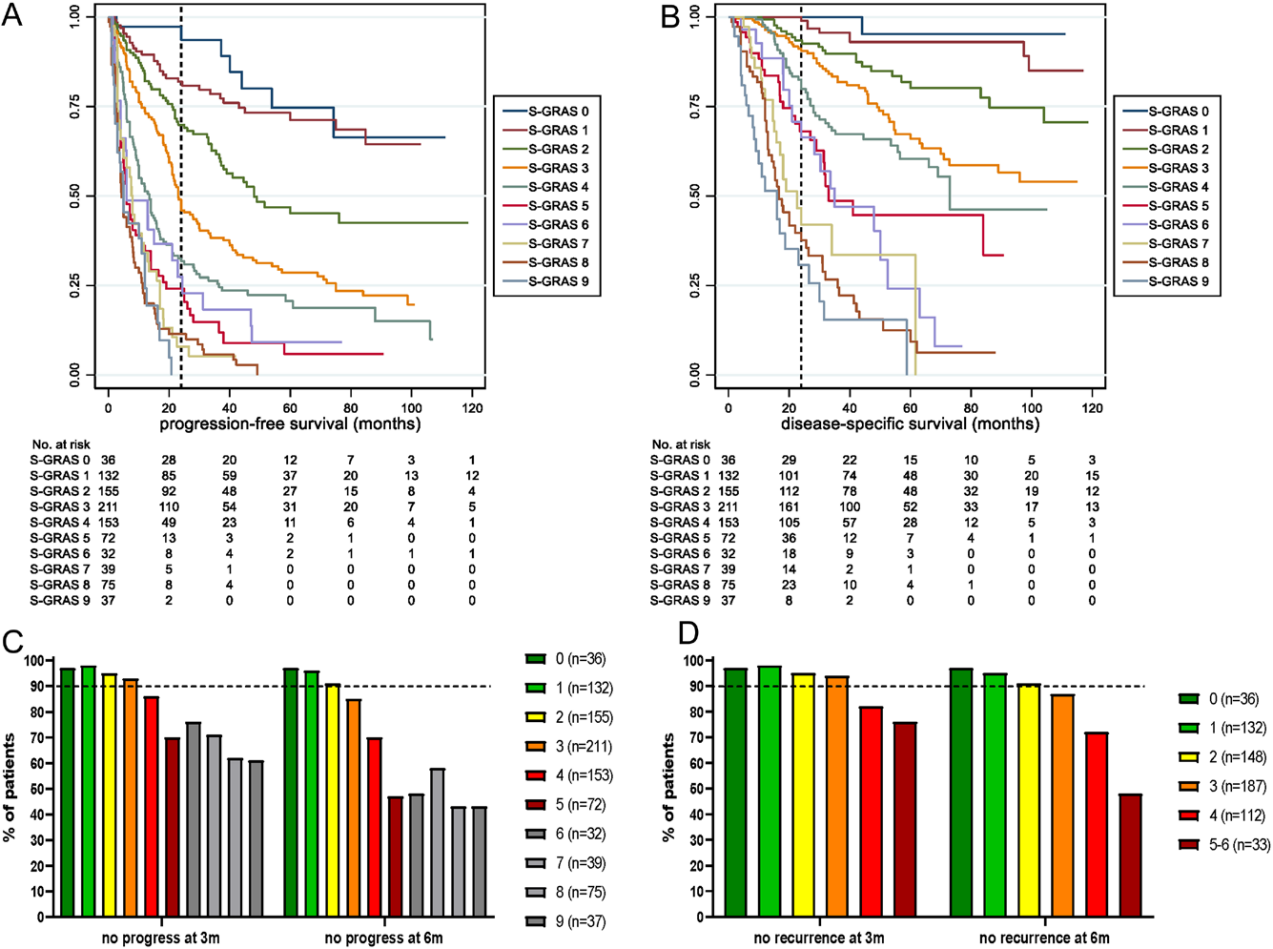
Prognostic role of the individual S-GRAS score categories (0–9). (A) Kaplan–Meier (KM) survival curves for progression-free survival; (B) KM survival curves for disease-specific survival. (C) Percentages of patients without documented disease progress at routine surveillance imaging after 3 and 6 months from primary surgery according to individual S-GRAS scores (*n* = 942). (D) Percentages of patients without documented disease recurrence (after complete resection, *n*  = 648) at routine surveillance imaging after 3 and 6 months from primary surgery according to individual S-GRAS scores. (C and D) Number of patients in each score group in brackets. Dotted line set at 90%.

Importantly, the ten individual S-GRAS scores (0–9) further stratified subgroups of patients with different clinical outcomes for PFS and DSS. KM plots and median survival data, percentage survival at 24 and 60 months as well as the HR values, 95% CI and *P*-values for PFS and DSS are reported in Fig. 3A, B, Table 1, and Supplementary Table 6. Briefly, clearer stratification was observed comparing S-GRAS 1 with 2 (DSS HR 2.1, PFS HR 1.9), 3 (DSS HR 3.6, PFS HR 3.3), 4 (DSS HR 5.7, PFS HR 5.1), and 5 (DSS HR 10.0, PFS HR 8.7). Patients with S-GRAS 0 (4%) showed a reduced risk of progression or death compared to S-GRAS 1 (PFS HR 0.7, DSS HR 0.3), though this was not statistically significant. Individual S-GRAS scores 6–9 did not provide clear stratification. Notably, discrimination statistics for the individual S-GRAS scores showed the highest C-index and R^2^_D_ statistic (PFS: C-index = 0.73, R^2^_D_ = 0.30, Table 1; DSS: C-index = 0.79, R^2^_D_ = 0.45, respectively, Supplementary Table 6).

To investigate the potential role of individual S-GRAS scores in guiding radiological surveillance, we assessed the percentage of ACC patients without disease progression or recurrence within the first 3 and 6 months after primary surgery. We observed the biggest differences in patients with S-GRAS 2, 3, 4, and 5 with an increasing frequency of disease progression in the first 6 months of surveillance (Fig. 3C and  D).

### Prognostic performance of S-GRAS on recurrence-free survival

Considering only patients with R0 status (*n* = 648), ENSAT staging, Ki67 index, and symptoms – but not age – represented significant prognostic factors at univariable analysis. Overall, the discriminative performance of S-GRAS (C-index = 0.67) remained superior to ENSAT staging (C-index = 0.62, *P* = 0.001), age (C-index = 0.52, *P* < 0.0001), symptoms (C-index = 0.57, *P* < 0.0001), and Ki67 0–9%, 10–19%, and ≥20% groups (C-index = 0.65, *P* = 0.052). The corresponding R^2^_D_ figures were 0.23, 0.11, 0.01, 0.08, and 0.18, respectively.

### Prognostic performance of S-GRAS and adjuvant mitotane

We included 795 patients eligible for this analysis; 481 adjuvant mitotane-treated (60.5%) and 314 untreated (39.5%) (Supplementary Table 7). Mitotane-treated patients more frequently presented with symptoms (70% vs 63%, *P* = 0.034) and had higher Ki67 index (≥20 in 50% vs 33%, *P* < 0.0001).

#### Recurrence-free survival

**Figure 4:**
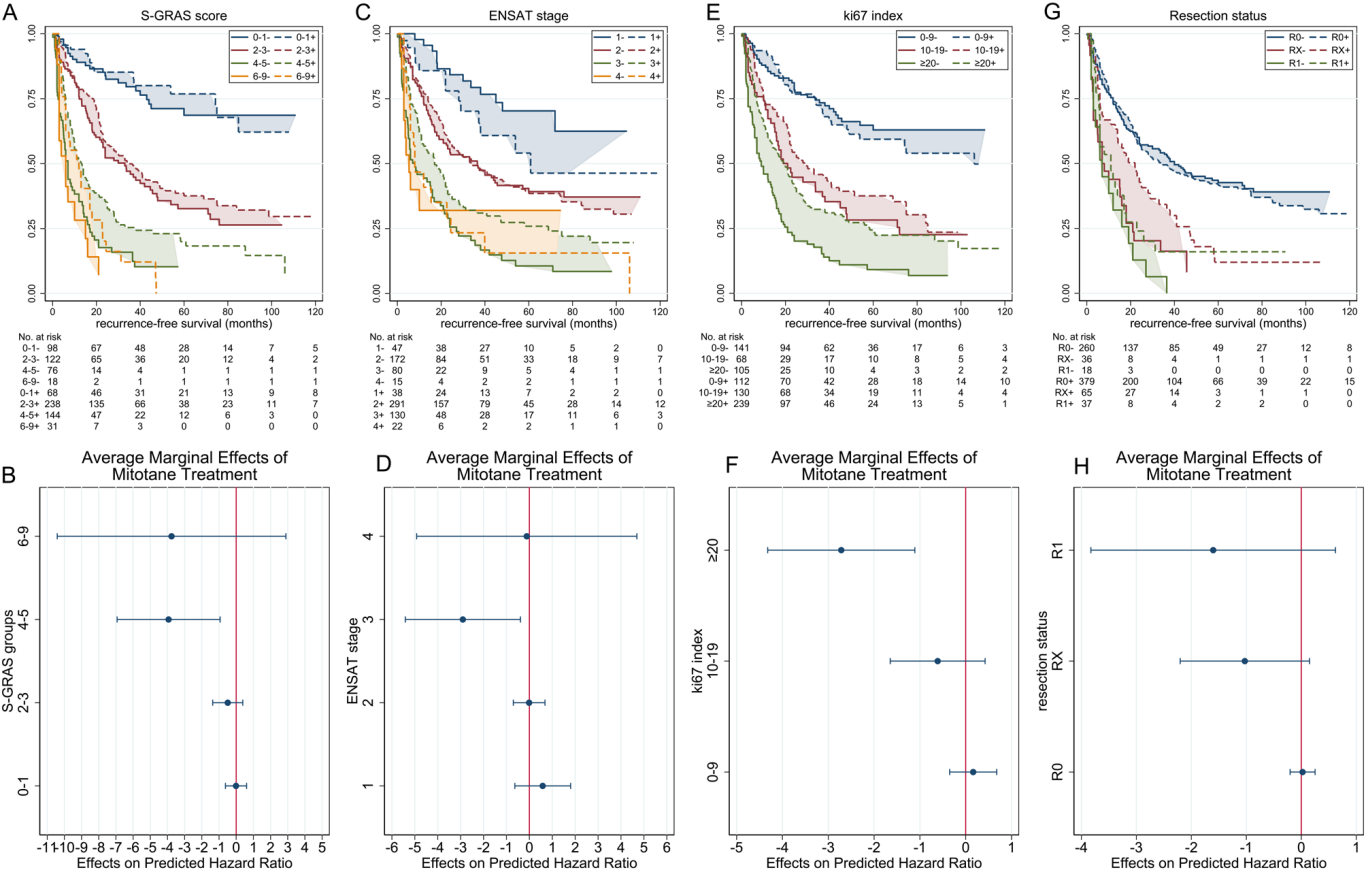
Kaplan–Meier (KM) curves depicting recurrence-free survival in patients untreated or treated with adjuvant mitotane; scatter plots showing the change in hazard ratio (marginal effects) as a result of treatment. According to (A and B) S-GRAS score groups, (C and D) ENSAT stage, (E and F) Ki67, and (G and H) resection status. ‘–’ and ‘+’ signs in the legends of the KM plots indicate no treatment and with treatment, respectively.

S-GRAS had superior discriminatory performance compared to its individual components in both mitotane-treated and -untreated cohorts. The C-index and R^2^_D_ statistics were higher for the S-GRAS groups compared to ENSAT stage, Ki67 index, and resection status (Table 2). Moreover, we compared the disease recurrence risk in treated and untreated patients in each S-GRAS group. Interestingly, there was a trend to reduction in the HR across the S-GRAS groups with mitotane treatment. This reduction was only statistically significant in S-GRAS 4–5 (*P* = 0.010), where HR fell from 9.3 (95% CI 5.8–15.0) to 5.4 (95% CI 3.4–8.4) (Fig. 4A, B and Table 2). Considering ENSAT staging, only the change at ENSAT stage 3 was statistically significant (from 7.1, 95% CI 4.0–13.0, to 4.2, 95% CI 2.3–7.5, *P =* 0.024) (Fig. 4C, D and Table 2). Regarding Ki67 index, only patients with Ki67 ≥20% showed a statistically significant reduction in HR with mitotane (from 6.0, 95% CI 4.2–8.8, to 3.4, 95% CI 2.4–4.7; *P=* 0.001, Fig. 4E, F and Table 2). No changes in HR at each resection status were statistically significant (Fig. 4G, H and Table 2).

**Table 2:** Univariate survival analysis: median recurrence-free survival (RFS), hazard ratios and discrimination analysis of single prognostic variables and S-GRAS scoring in patients with adrenocortical carcinoma treated or not treated with mitotane (MT) adjuvant (*n* = 795).

Variables/MT status	*n*	Median RFS, months (95% CI)	HR (95% CI)	*P*-value (HR)	HR difference – untreated vs treated (95% CI)	*P*-value (difference)	Harrell’s C-index (95% CI)	Royston–Sauerbrei’s R^2^_D_ statistic
ENSAT stage (MT = No)							0.67 (0.63, 0.71)	0.18 (0.11, 0.26)
1	47	NR	1	Reference				
2	172	36.5 (21.0, 45.6)	2.6 (1.5, 4.7)	0.001				
3	80	8.0 (5.0, 16.0)	7.1 (3.9, 12.9)	<0.0001				
4	15	5.3 (2.9, NR)	6.4 (2.9, 14.3)	<0.0001				
ENSAT stage (MT = Yes)							0.60 (0.56, 0.63)	0.07 (0.03, 0.13)
1	38	60.9 (29.0, NR)	1.6 (0.7, 3.4)	0.242	0.58 (−0.63, 1.80)	0.348		
2	291	34.0 (26.0, 48.0)	2.6 (1.5, 4.6)	0.001	−0.01 (−0.70, 0.68)	0.976		
3	130	17.0 (11.0, 22.0)	4.2 (2.3, 7.5)	<0.0001	−2.90 (−5.42, −0.39)	0.024		
4	22	8.0 (5.7, 24.4)	6.3 (3.0, 12.9)	<0.0001	−0.11 (−4.92, 4.69)	0.963		
Ki67 index (MT = No)							0.70 (0.66, 0.74)	0.32 (0.23, 0.41)
0–9	141	199.8 (NR, NR)	1	Reference				
10–19	68	21.0 (15.2, 36.9)	2.9 (1.9, 4.5)	<0.0001				
≥20	105	9.0 (6.0, 14.0)	6.1 (4.2, 8.8)	<0.0001				
Ki67 index (MT=Yes)							0.62 (0.59, 0.65)	0.12 (0.06, 0.19)
0–9	112	106.1 (49.0, NR)	1.2 (0.8, 1.8)	0.504	0.16 (−0.35, 0.68)	0.535		
10–19	130	29.0 (22.0, 44.5)	2.3 (1.6, 3.4)	<0.0001	−0.61 (−1.65, 0.42)	0.246		
≥20	239	19.7 (14.0, 23.0)	3.4 (2.4, 4.8)	<0.0001	−2.72 (−4.32, −1.11)	0.001		
Resection status (MT= No)							0.59 (0.56, 0.62)	0.18 (0.09, 0.27)
R0	260	40.0 (30.0, 60.0)	1	Reference				
RX	36	7.0 (3.0, 16.0)	2.8 (1.9, 4.3)	<0.0001				
R1	18	7.0 (2.9, 16.0)	4.1 (2.4, 6.9)	<0.0001				
Resection status (MT= Yes)							0.57 (0.54, 0.59)	0.09 (0.04, 0.16)
R0	379	34.0 (25.0, 48.9)	1.0 (0.8, 1.3)	0.836	0.02 (−0.20, 0.25)	0.837		
RX	65	22.0 (13.0, 29.0)	1.8 (1.3, 2.5)	0.001	−1.03 (−2.20, 0.15)	0.087		
R1	37	11.0 (6.0, 17.0)	2.47 (1.6, 3.7)	<0.0001	−1.60 (−3.83, 0.62)	0.158		
S-GRAS group (MT = No)							0.72 (0.69, 0.76)	0.30 (0.22, 0.38)
0–1	98	199.8 (199.8, NR)	1	Reference				
2–3	122	33.0 (21.6, 44.6)	3.1 (1.9, 4.9)	<0.0001				
4–5	76	7.0 (5.0, 10.0)	9.3 (5.8, 14.9)	<0.0001				
6–9	18	6.0 (2.9, 15.0)	11.6 (6.1, 21.9)	<0.0001				
S-GRAS group (MT = Yes)							0.66 (0.63, 0.69)	0.18 (0.12, 0.25)
0–1	68	181.6 (75.0, NR)	1.0 (0.5, 1.8)	0.976	−0.01 (−0.63, 0.61)	0.975		
2–3	238	34.0 (25.0, 48.0)	2.6 (1.7, 4.1)	<0.0001	−0.49 (−1.37, 0.38)	0.271		
4–5	144	13.5 (9.8, 16.5)	5.4 (3.4, 8.4)	<0.0001	−3.94 (−6.93, −0.94)	0.010		
6–9	31	12.0 (6.0, 17.0)	7.9 (4.5, 13.8)	<0.0001	−3.76 (−10.42, 2.89)	0.268		

HR, hazard ratio; NR, not reached (= median survival not reached, that is, percentage survival remained >50% so this value and/or its 95% CIs cannot be computed).

#### Disease-specific survival

The C-index and R^2^_D_ statistics were again higher for the S-GRAS groups compared to ENSAT stage, Ki67 index, and resection status. Supplementary Fig. 2 and Supplementary Table 8 summarises the marginal effects of mitotane treatment for disease-related death. Despite trending HR changes as a result of covariate change from untreated to treated with adjuvant mitotane, none were statistically significant.

## Discussion

This is, to our knowledge, the largest multicentre study on comprehensive prognostic stratification in ACC patients based on clinical/histopathological characteristics, which enabled us to accurately assess the prognostic performance of the newly proposed S-GRAS score (19). Previous studies suggested promising prognostic biomarkers at the tumour level (19, 25, 26, 27, 28). However, molecular analysis is not routine practice in ACC. Contrarily, the S-GRAS components are available as part of standard clinical practice for operated ACC patients.

We demonstrated superior prognostic discrimination for both DSS and PFS by the S-GRAS score compared to ENSAT staging (3) and Ki67 index (9), the current standard ACC prognostic tools (6). Moreover, the ten individual S-GRAS score values showed even better identification of further subgroups of patients with different clinical outcome. Hereby, it is worth mentioning that not all individual S-GRAS components carry the same weight – with some (e.g. ENSAT stage and Ki67) having a stronger influence on PFS/DSS compared to others.

Few recent studies proposed a combination of clinical/histopathological characteristics to improve prognostication in ACC (20, 29) and the prognostic role of different combinations based on mathematical models has also been reported (30, 31, 32, 33, 34). However, these studies included small cohorts of patients and/or heterogeneous variables or outcomes and/or did not include a comparative statistics, Supplementary Table 9.

The modified version of ENSAT stage (mENSAT) (8) was not considered in the present study due to the selection bias that excluded non-operable patients. Future studies are therefore needed to investigate the added value of GRAS components to mENSAT classification in non-resectable disease.

Mitotane is the mainstay adjuvant therapy in ACC, which can carry significant toxicity. Mitotane use is based on retrospective and conflicting evidence (4, 5, 21, 35, 36, 37, 38), and there are no reliable markers to predict treatment response (20). Currently, adjuvant mitotane is proposed for patients considered at high risk of recurrence (Ki67 index ≥10% and/or resection status RX-R1, and/or ENSAT stage 3 and 4) (6, 10). Results of the first prospective study on mitotane adjuvant in low-risk patients are still awaited (ADIUVO, ClinicalTrials.gov identifier 777244). Here, we firstly show that only patients with Ki67 index ≥20% and/or ENSAT stage 3 had a significant longer RFS if treated with adjuvant mitotane – independently from the resection status. Moreover, S-GRAS seems to best stratify patients with different outcomes whether patients received mitotane or not, with S-GRAS score values 4–5 being associated with longer RFS in mitotane-treated patients. Therefore, we hereby hypothesise that S-GRAS can be used also to stratify patients more likely to benefit from adjuvant mitotane.

**Figure 5:**
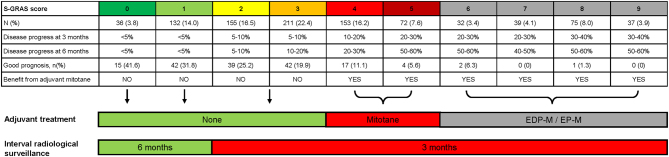
Proposal for the potential use of S-GRAS score categories in the clinical practice. Table showing frequency of individual S-GRAS scores, percentage of patients with disease progression at surveillance radiological imaging at 3 and 6 months after primary surgery, percentage of patients with favourable prognosis and potential benefit from adjuvant mitotane treatment according to the findings of the present paper. Patients were defined to have a favourable prognosis by progression-free survival ≥ 24 months and disease-specific survival ≥ 60 months (19). Suggestion for the potential use of S-GRAS categories in the clinical decision-making process: (A) adjuvant treatment with mitotane or cytotoxic drugs plus mitotane (i.e. EDP-M, Etoposide-Doxorubicine-CisPlatin or EP-M, Etoposide-CisPlatin); (B) interval of radiological surveillance.

We hereby propose a novel, improved management strategy for operated ACC patients, focusing on S-GRAS scoring, as compared to the current European guideline (Fig. 5 and Supplementary Table 10) (6). First, we suggest that S-GRAS 0–1 patients (18%) might be offered longer radiological surveillance intervals (e.g. every 6 instead of the 3 months recommended by current guidelines) (6, 10). This may reduce radiation exposure to patients with a low likelihood of recurrence. Second, patients with S-GRAS 0–3 seem less likely to benefit from adjuvant mitotane and could be saved from this treatment (avoiding unnecessary adverse effects). However, specifically in S-GRAS group 2–3, the direct role of Ki67 on effects of adjuvant mitotane needs to be further validated. We also hypothesise that S-GRAS score 6–9 may better pre-select higher risk ACC patients suited for a more aggressive adjuvant approach such as cytotoxic chemotherapy. In fact, a very recent retrospective study suggests that patients with very high risk for recurrence benefit from adjuvant chemotherapy with a platinum-based therapy (39). Furthermore, this approach is currently under investigation in prospective trials (ADIUVO-2 trial, ClinicalTrials.gov identifier NCT03583710, and ACACIA trial, NCT03723941).

Limitations of this study comprise its retrospective nature and the non-centralised radiological and histopathological reporting. Like all prognostic studies, the indirect impact of additional adjuvant treatments (i.e. local radiotherapy) or therapeutic interventions (i.e. subsequent local therapies, cytotoxic drugs) on overall survival cannot be excluded. However, our study’s major strengths, that is, the unique, well clinically annotated and vast patient cohort, standardised method of data extraction from ENSAT registry, and expert statistical analysis, allowed robust conclusions in this rare cancer.

In conclusion, our findings on a large cohort of ACC patients demonstrate that S-GRAS scoring can improve the management of ACC, personalising the frequency of radiological surveillance and rationalising the use of adjuvant mitotane after radical surgery. This S-GRAS-based strategy now requires validation in future prospective studies aimed to compare the prognostic role of S-GRAS (for predicting disease recurrences and response to mitotane treatment) before being implemented in clinical practice.

## Supplementary Material

Supplementary Figure 1

Supplementary Figure 2

Suppl Table 1. Details about recruitment process, data collection, modalities of disease surveillance and details on mitotane treatment.

Suppl Table 2. Calculation of S-GRAS score starting from five baseline clinical and histopathological characteristics

Suppl Table 3: Sample size calculation

Suppl Table 4. Demographics of the entire cohort of patients with adrenocortical carcinoma (n=942). Data collected at the time of diagnosis

Supplementary Table 5 – Multivariable Cox Regression model for progression-free survival (A) and disease-specific survival (B) with and without resection status, age and symptoms (RAS components) added to ENSAT tumour stage (S) and ki67 index (G).

Suppl Table 6. Univariate survival analysis: median disease-specific survival (DSS), percentage survival at 24 and 60 months, hazard ratios and discrimination analysis of single prognostic variables and S-GRAS scoring.

Suppl Table 7. Demographic data for patients with adrenocortical carcinoma in the adjuvant mitotane cohort (n=795)

Suppl Table 8. Univariate survival analysis: median disease-specific survival (DSS), hazard ratios and discrimination analysis of single prognostic variables and S-GRAS scoring in patients with adrenocortical carcinoma treated or not treated with mitotane adjuvant (n=795).

Suppl Table 9. Overview of different available models for prognostication in patients with ACC compared to the present series.

Supplementary Table 10. Overview of our hypothetically proposed “S-GRAS-based recommendation” as compared with the current European ACC Guidelines (Fassnacht et al EJE 2018).

## Declaration of interest

A J Sitch, W Arlt, and M. Fassnacht are on the editorial board of EJE. A J Sitch, W Arlt, and M Fassnacht were not involved in the review or editorial process for this paper, on which they are listed as an author.

## Funding

This study was supported by the following funding sources: Deutsche Forschungsgemeinschaft http://dx.doi.org/10.13039/501100001659 (DFG) within the CRC/Transregio (314061271 – TRR 205 to M F and M K), project FA-466/8-1 and RO-5435/3-1 (to M F, M K, and C L R, respectively), and project FA-466/4-2 (to M F), the Deutsche Krebshilfe http://dx.doi.org/10.13039/501100005972 (70113526 to M F and C L R), Fundacao de Amparo a Pesquisa do Estado de Sao Paulo (FAPESP) to M C B V F (2017/26345-5). This paper presents independent research supported by the NIHR Birmingham Biomedical Research Centre at the University Hospitals http://dx.doi.org/10.13039/100012324 Birmingham NHS Foundation Trust and the University of Birmingham. The views expressed are those of the author(s) and not necessarily those of the NHS, the NIHR or the Department of Health and Social Care (S B, A S, J D, and W A).
