# Supplementary figures and images for: S-GRAS score for prognostic classification of adrenocortical carcinoma: an international, multicenter ENSAT study
Source: Eur J Endocrinol. 2021 Oct 27;186(1):25–36. doi: 10.1530/EJE-21-0510 (PMC8679848; doi:10.1530/EJE-21-0510)

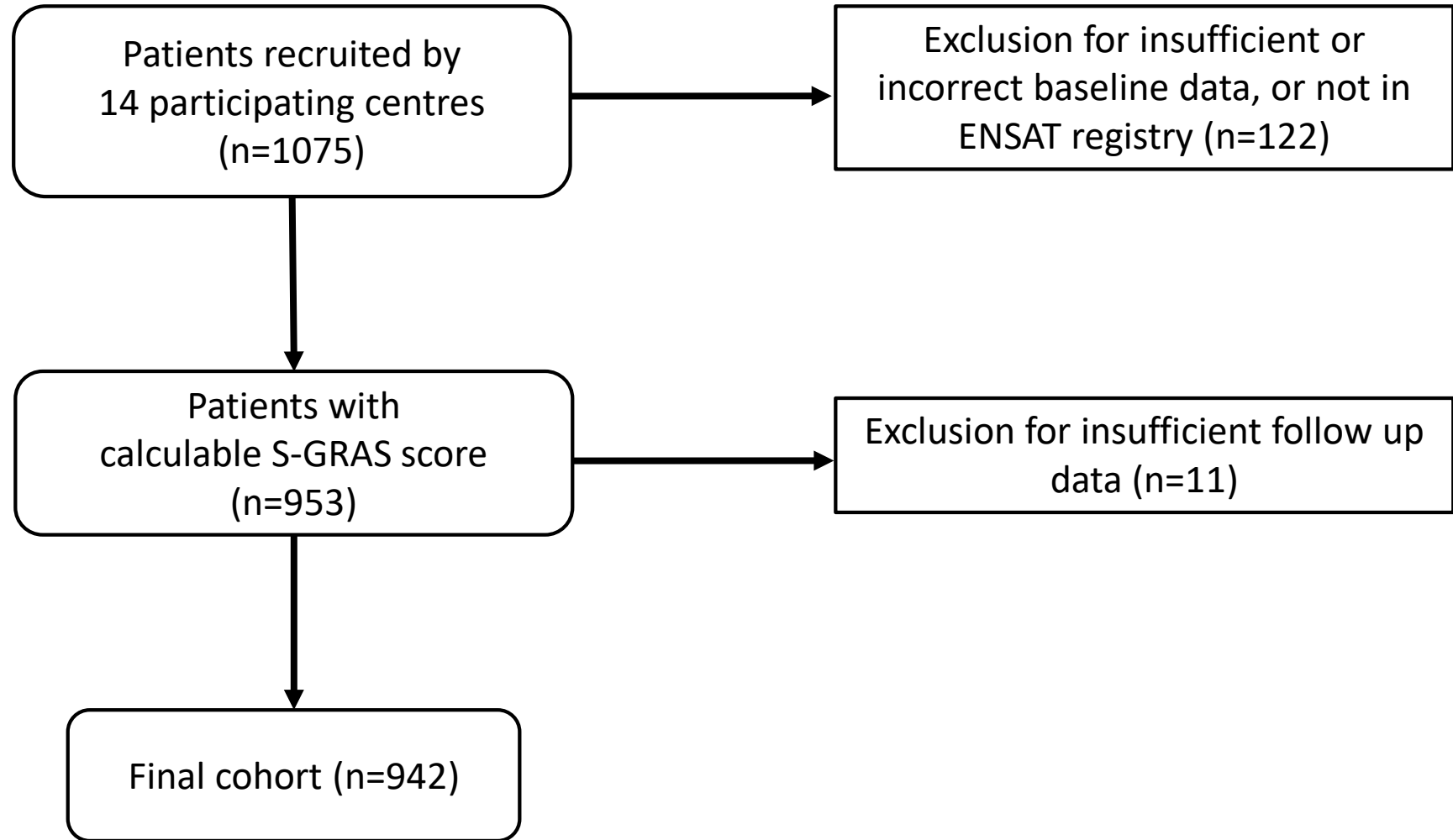

Supplement: Supplementary Figure 1 [file supplementary_figure_1.pdf]

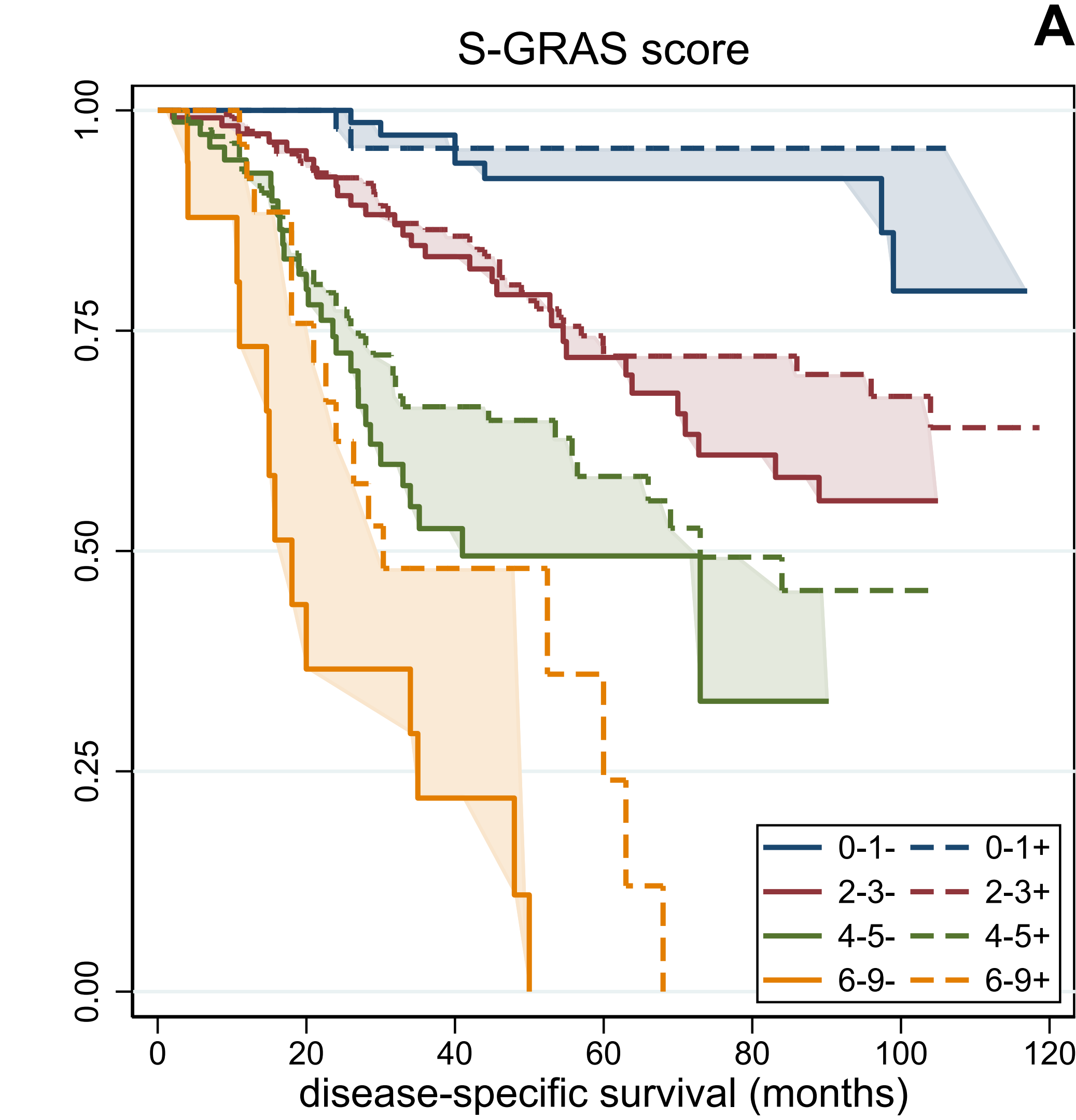

|             |     |     |     |    |    |    |    |
|-------------|-----|-----|-----|----|----|----|----|
| No. at risk |     |     |     |    |    |    |    |
| 0-1-        | 98  | 77  | 61  | 37 | 22 | 12 | 6  |
| 2-3-        | 122 | 97  | 62  | 37 | 25 | 14 | 12 |
| 4-5-        | 76  | 47  | 17  | 11 | 3  | 2  | 2  |
| 6-9-        | 18  | 6   | 3   | 0  | 0  | 0  | 0  |
| 0-1+        | 68  | 53  | 35  | 26 | 18 | 13 | 12 |
| 2-3+        | 238 | 172 | 115 | 63 | 40 | 22 | 13 |
| 4-5+        | 144 | 92  | 52  | 24 | 13 | 4  | 2  |
| 6-9+        | 31  | 18  | 7   | 3  | 0  | 0  | 0  |

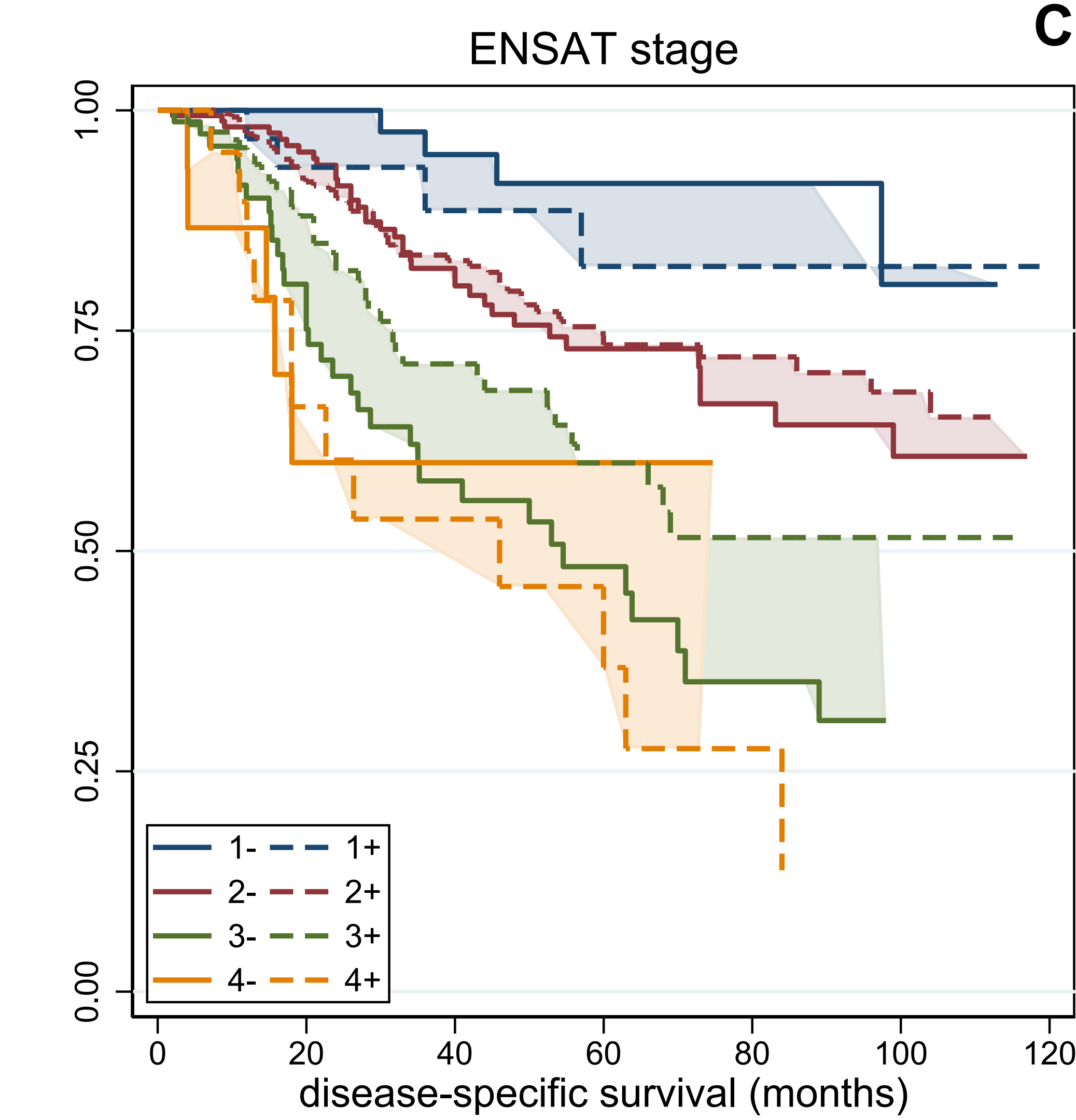

|             |     |     |     |    |    |    |    |
|-------------|-----|-----|-----|----|----|----|----|
| No. at risk |     |     |     |    |    |    |    |
| 1-          | 47  | 44  | 33  | 15 | 11 | 6  | 3  |
| 2-          | 172 | 130 | 82  | 52 | 29 | 17 | 12 |
| 3-          | 80  | 47  | 26  | 16 | 10 | 5  | 5  |
| 4-          | 15  | 6   | 2   | 2  | 0  | 0  | 0  |
| 1+          | 38  | 27  | 18  | 12 | 5  | 3  | 1  |
| 2+          | 291 | 211 | 132 | 72 | 47 | 26 | 20 |
| 3+          | 130 | 86  | 52  | 27 | 17 | 9  | 5  |
| 4+          | 22  | 11  | 7   | 5  | 2  | 1  | 1  |

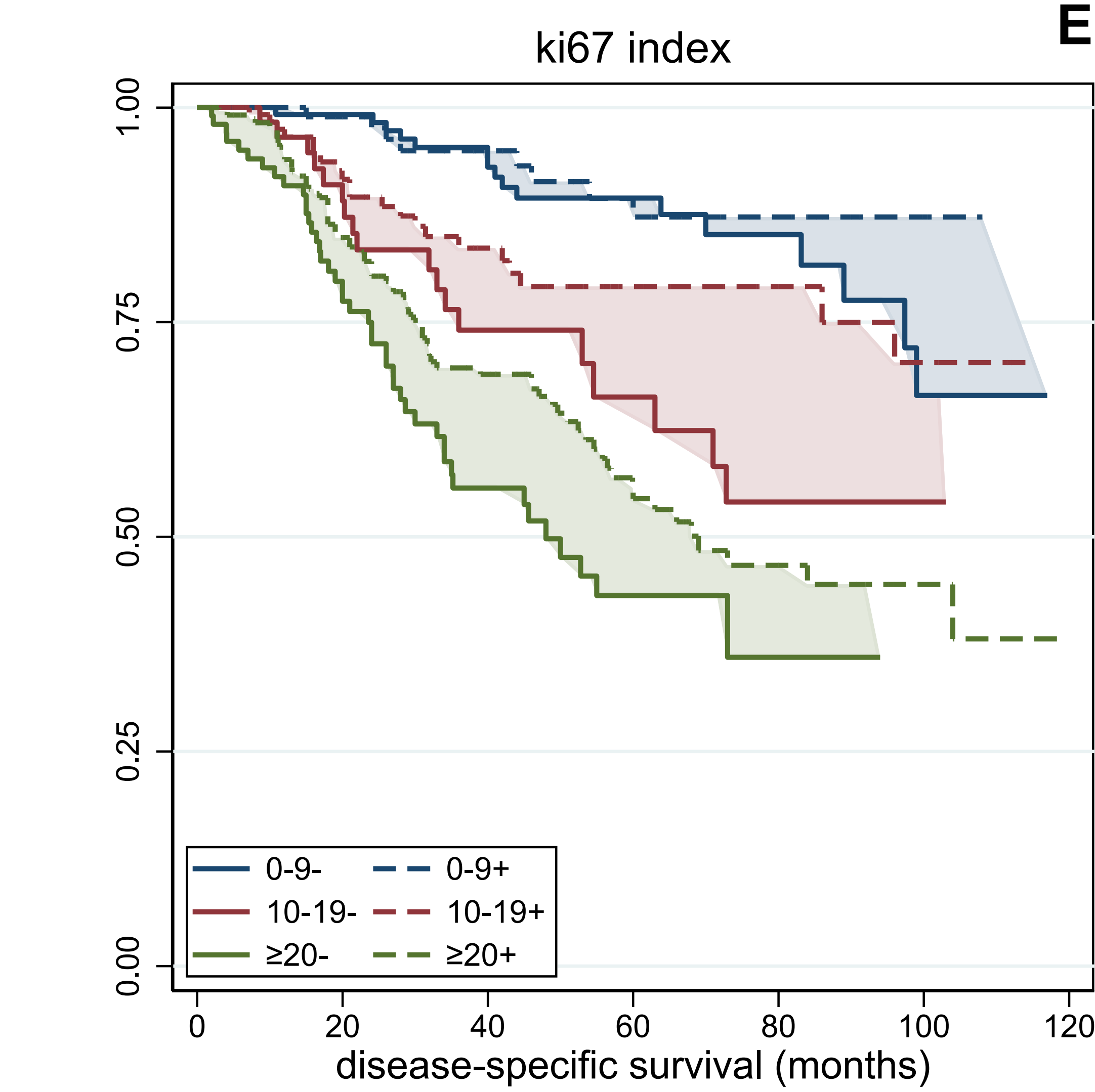

|             |     |     |    |    |    |    |    |
|-------------|-----|-----|----|----|----|----|----|
| No. at risk |     |     |    |    |    |    |    |
| 0-9-        | 141 | 111 | 83 | 52 | 28 | 12 | 5  |
| 10-19-      | 68  | 48  | 28 | 17 | 13 | 9  | 8  |
| ≥20-        | 105 | 68  | 32 | 16 | 9  | 7  | 7  |
| 0-9+        | 112 | 85  | 58 | 41 | 28 | 18 | 15 |
| 10-19+      | 130 | 90  | 59 | 30 | 21 | 13 | 9  |
| ≥20+        | 239 | 160 | 92 | 45 | 22 | 8  | 3  |

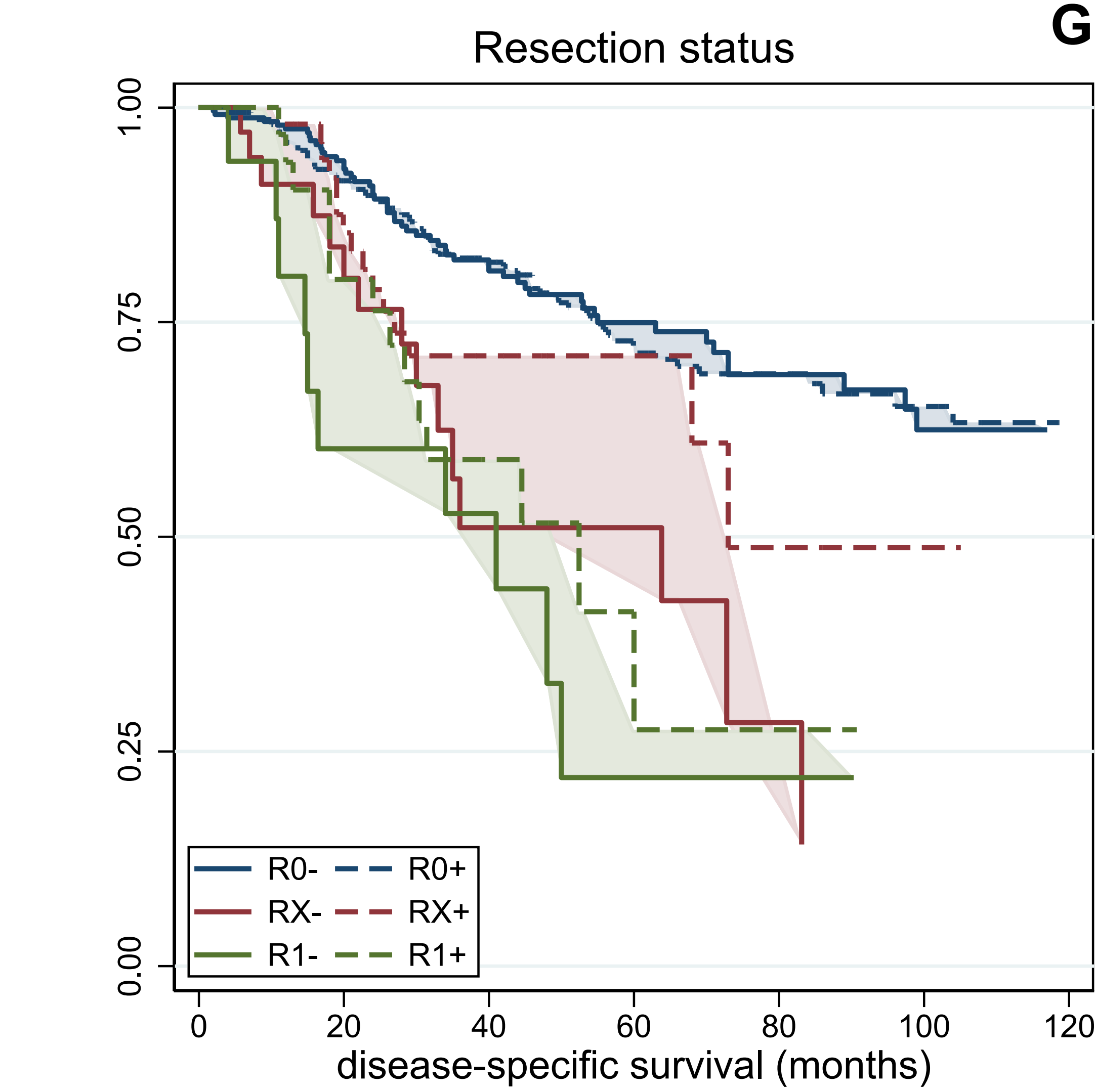

|             |     |     |     |     |    |    |    |
|-------------|-----|-----|-----|-----|----|----|----|
| No. at risk |     |     |     |     |    |    |    |
| R0-         | 260 | 196 | 129 | 77  | 46 | 26 | 18 |
| RX-         | 36  | 23  | 8   | 6   | 2  | 1  | 1  |
| R1-         | 18  | 8   | 6   | 2   | 2  | 1  | 1  |
| R0+         | 379 | 272 | 174 | 102 | 65 | 38 | 27 |
| RX+         | 65  | 40  | 25  | 11  | 4  | 1  | 0  |
| R1+         | 37  | 23  | 10  | 3   | 2  | 0  | 0  |

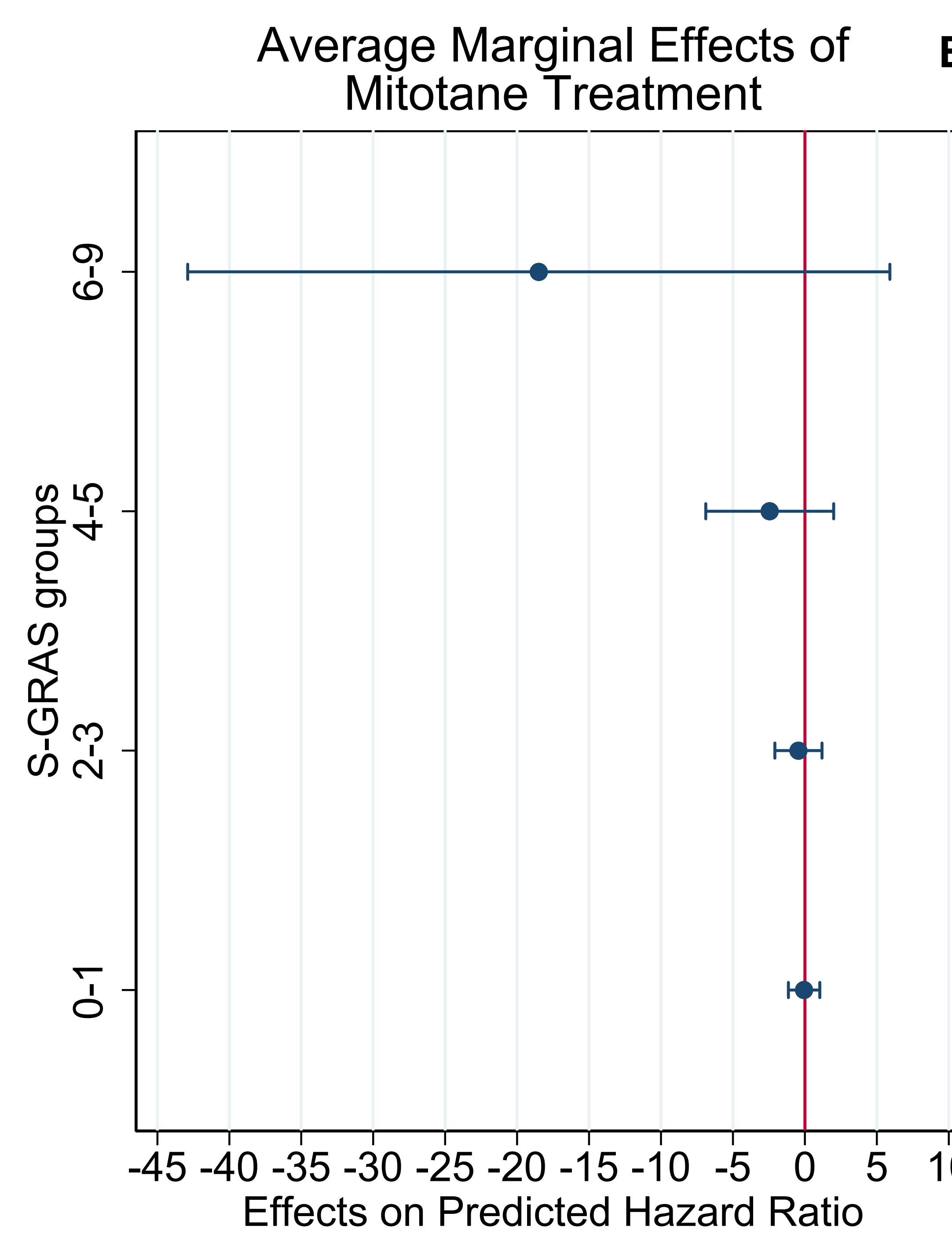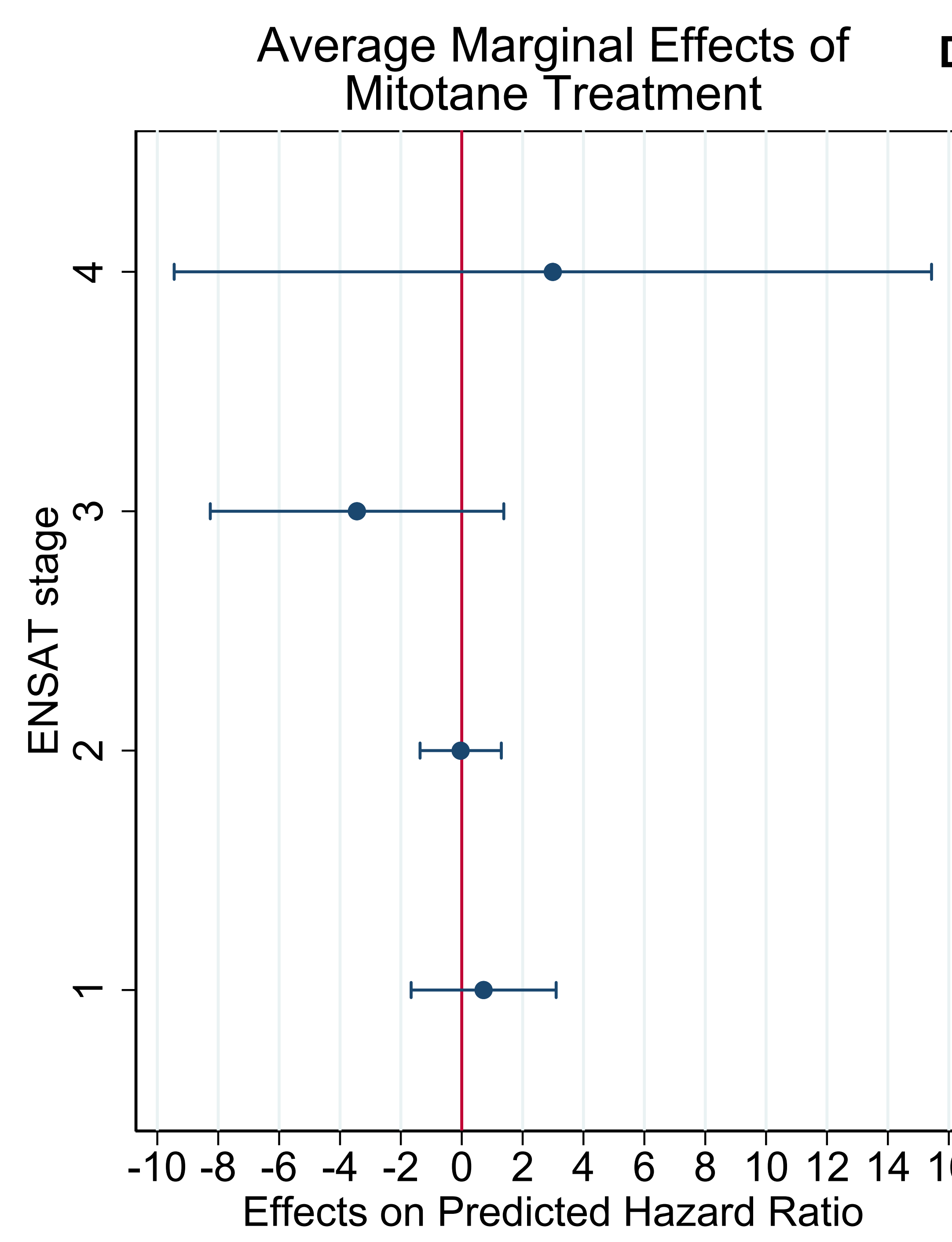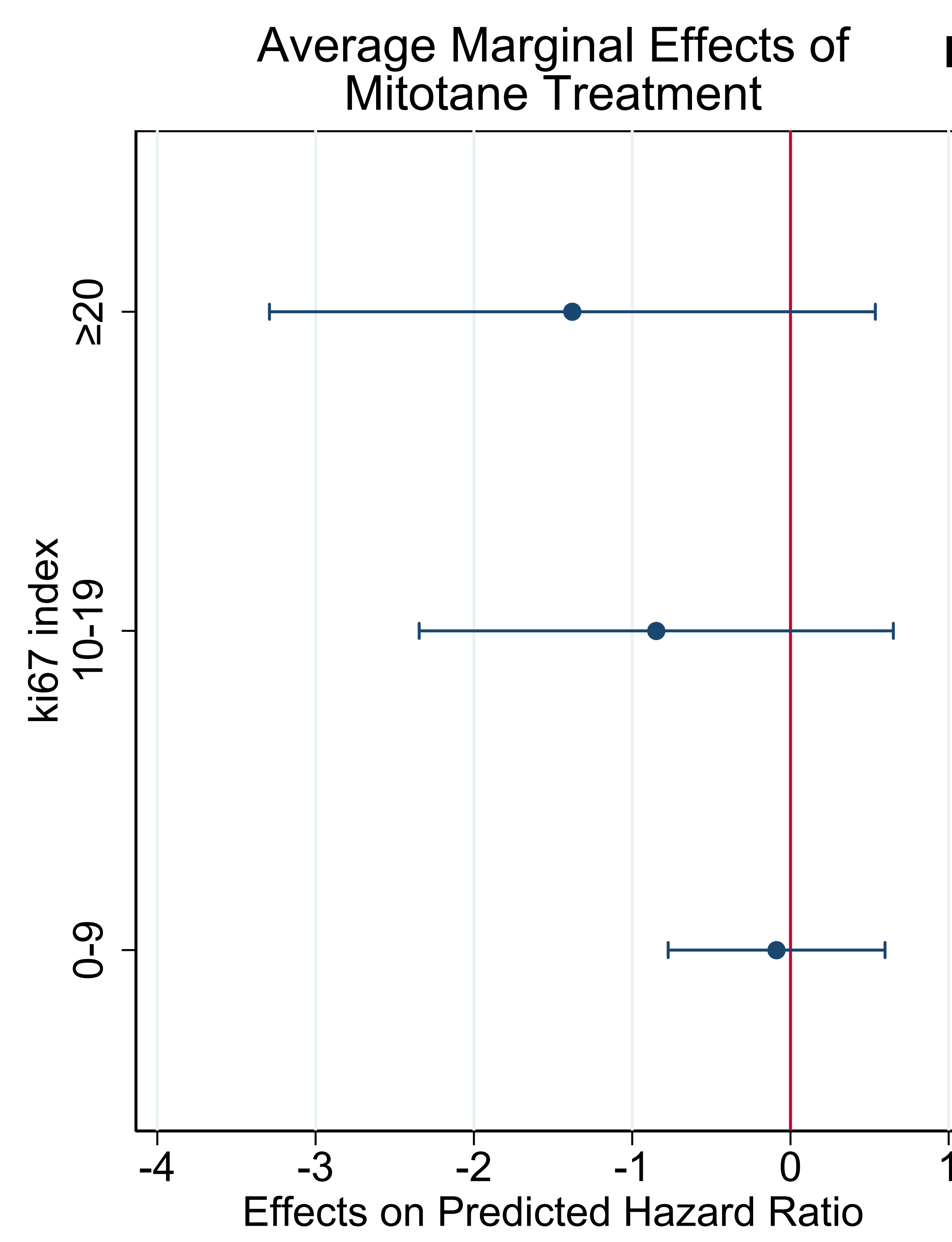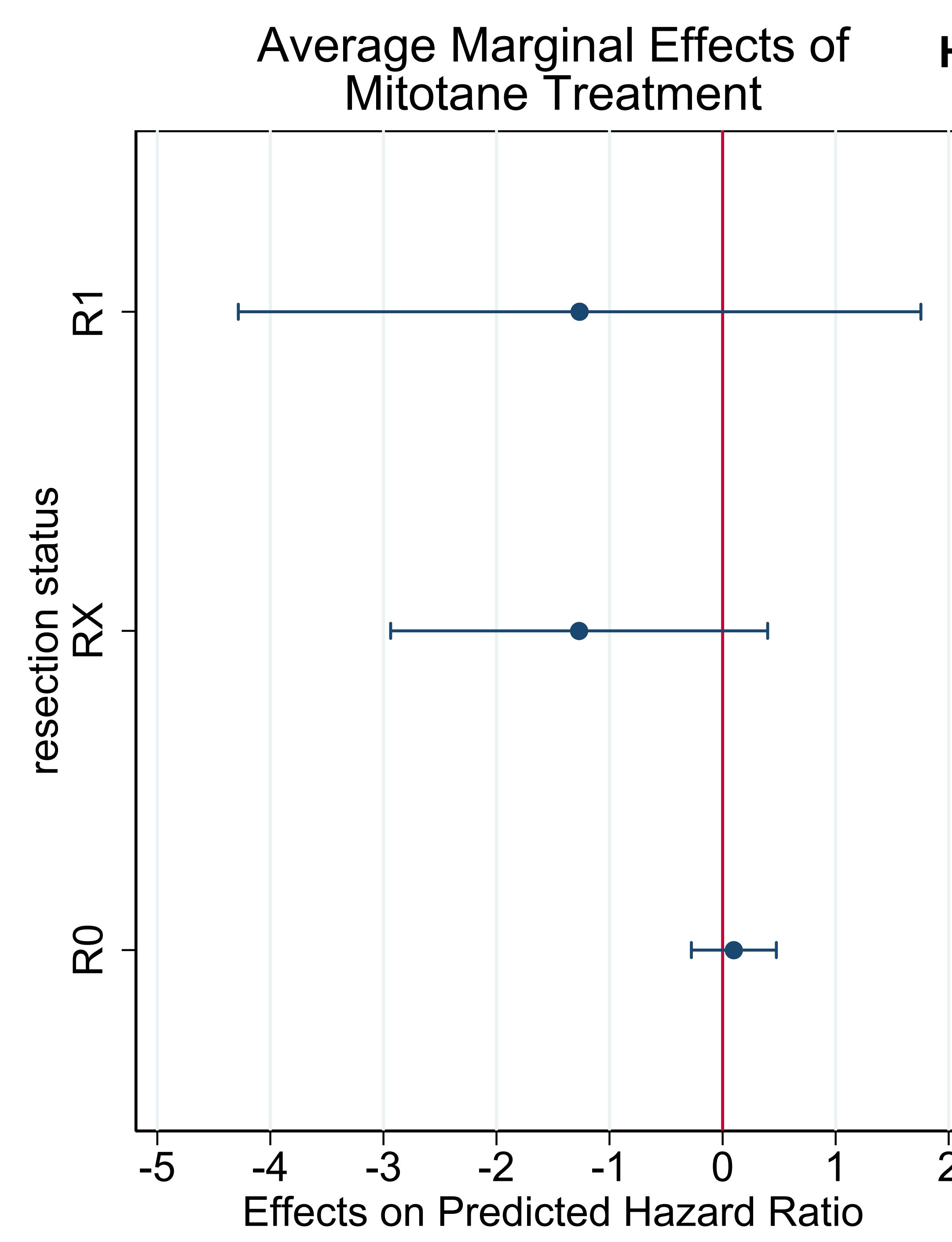

Supplement: Supplementary Figure 2 [file supplementary_figure_2.pdf]
