# Supplementary material for: S-GRAS score for prognostic classification of adrenocortical carcinoma: an international, multicenter ENSAT study
Source: Eur J Endocrinol. 2021 Oct 27;186(1):25–36. doi: 10.1530/EJE-21-0510 (PMC8679848; doi:10.1530/EJE-21-0510)
Supplement: Suppl Table 1. Details about recruitment process, data collection, modalities of disease surveillance and details on mitotane treatment. [file supplementary_table_1.pdf]

Suppl Table 1. Details about recruitment process, data collection, modalities of disease surveillance and details on mitotane treatment.

**Recruitment process:** The present study proposal and data collection sheet were approved by the ENSAT ACC Working Group (www.ensat.org). The study was advertised to all ENSAT members and 14 centres in 9 countries agreed to participate. The participants were recruited through specialist centres within the ENSAT and registered in the ENSAT database (www.ensat.org) (see below).
**List of participating Institutions:** University of Birmingham - UK, University Hospital of Würzburg - Germany, ASST-Spedali Civili Brescia - Italy, University of Turin San Luigi Hospital - Italy, Diabète et Nutrition CHU de Bordeaux - France, University Hospital Centre Zagreb - Croatia, Hospital das Clinicas da Universidade de São Paulo - Brazil, Cochin Hospital Paris - France, Gustave Roussy Cancer Center Paris - France, Máxima MC Eindhoven - Netherlands, Theagenio Cancer Hospital Thessaloniki - Greece, University of Florence - Italy, Clinica Polispecialistica San Carlo Milano - Italy, Beaumont Hospital Dublin - Republic of Ireland.
**ENSAT Registry and Ethics:** All participating centres hold locally approved ethics for including patients with adrenal tumours in the ENSAT registry. Inclusion in the ENSAT registry comprises entry of clinical data in a standardised format for use in any current and future adrenal tumour-related research approved by the ENSAT working groups; thus, no project-specific ethics had to be obtained.
**Data collection:** Patients were consecutively recruited and data were collected retrospectively. Date for completion of data collection was July 31, 2019. Data were recorded in a pseudonymised form in the ENSAT registry database (www.ensat.org). In case of missing or discrepant data, queries were raised and resolved with the participating centres.
**Modalities of disease surveillance:** Disease monitoring was performed by periodical testing of radiological cross-sectional imaging of the chest, abdomen and pelvis. According to the local routine, and more recently the ESE-ENSAT guideline (1), the radiological surveillance was as follows: a) after complete resection, every 3 months for two years, then every 3–6 months for a further three years (further follow-up imaging suggested up to ten years but was individually adapted); b) for advanced disease, the interval was generally every 3 months but could be modified based on prognostic factors (i.e. ENSAT stage and Ki67 index), general condition of the patient, expected treatment efficacy and treatment-related toxicity, as well as the available alternative treatment options.

Details of adjuvant treatment: Adjuvant treatment with mitotane (1,1-dichlorodiphenildichloroethane, o,p'-DDD, 500 mg oral tablets) was used in a subset of patient after radical surgery (16). Mitotane treatment was initiated at the discretion of each participating centre, informed by the literature (16) and more recently by the European Society for Endocrinology (ESE)-ENSAT clinical guideline (1), and following discussion with the patients. Modalities of mitotane treatment, such as initial high- or low-dose scheme and dose titration according to plasma mitotane concentrations (therapeutic window 14-20 mg/L) were chosen by the local endocrinologists according to local practice (18).
