# Supplementary material for: S-GRAS score for prognostic classification of adrenocortical carcinoma: an international, multicenter ENSAT study
Source: Eur J Endocrinol. 2021 Oct 27;186(1):25–36. doi: 10.1530/EJE-21-0510 (PMC8679848; doi:10.1530/EJE-21-0510)
Supplement: Suppl Table 2. Calculation of S-GRAS score starting from five baseline clinical and histopathological characteristics [file supplementary_table_2.pdf]

Suppl Table 2. Calculation of S-GRAS score starting from five baseline clinical and histopathological characteristics.

S-GRAS components	Group	Points
ENSAT tumour stage	1-2	0
	3	1
	4	2
Grading (according to ki67 index)	0-9%	0
	10-19%	1
	≥20%	2
Resection status	R0	0
	RX	1
	R1	2
	R2	3
Age	<50 yrs	0
	≥50 yrs	1
Symptoms	No	0
	Yes	1
