# Supplementary material for: S-GRAS score for prognostic classification of adrenocortical carcinoma: an international, multicenter ENSAT study
Source: Eur J Endocrinol. 2021 Oct 27;186(1):25–36. doi: 10.1530/EJE-21-0510 (PMC8679848; doi:10.1530/EJE-21-0510)
Supplement: Suppl Table 3: Sample size calculation [file supplementary_table_3.pdf]

**Suppl Table 3: Sample size calculation**

In the absence of any suitable D value for the S-GRAS score from previous literature, we used a number of possible values including the suggested value of D=1.4, which is corresponds to Harrell's C index of 0.73. The table shows a range of sample sizes required for a validation study to detect a difference of 0.4, at alpha level 0.05 (two-sided) and 90% power for the PFS and DSS endpoints. The censoring proportion for the former and latter were 0.39 and 0.70 respectively.

| Endpoint | D=1.4 (Harrell's C = 0.73) | D=1.5 (Harrell's C = 0.75) | D=1.6 (Harrell's C = 0.76) | D=1.7 (Harrell's C = 0.77) | D=1.8 (Harrell's C = 0.78) |
|---|---|---|---|---|---|
| PFS | 463 | 492 | 522 | 555 | 589 |
| DSS | 754 | 794 | 837 | 884 | 937 |

PFS = progression-free survival; DSS = disease-specific survival.
