# Supplementary material for: S-GRAS score for prognostic classification of adrenocortical carcinoma: an international, multicenter ENSAT study
Source: Eur J Endocrinol. 2021 Oct 27;186(1):25–36. doi: 10.1530/EJE-21-0510 (PMC8679848; doi:10.1530/EJE-21-0510)
Supplement: Suppl Table 4. Demographics of the entire cohort of patients with adrenocortical carcinoma (n=942). Data collected at the time of diagnosis [file supplementary_table_4.pdf]

Suppl Table 4. Demographics of the entire cohort of patients with adrenocortical carcinoma (n=942). Data collected at the time of diagnosis.

Variable	Value
**Age, years**	50 (38, 61)
**Male, n(%)**	359 (38)
**Symptoms, n(%)**	665 (71)
**Symptom type, n(%)**	
None	277 (29)
Tumour mass	175 (19)
Steroids	386 (41)
Cancer-related	40 (4)
Combinations of the above	64 (7)
**Hormone pattern, n(%)**	
Non-functional	360 (38)
Glucocorticoid excess	202 (21)
Androgen excess	80 (8)
Mineralocorticoid excess	25 (3)
Combinations of the above or other	196 (21)
Unknown	79 (8)
**ENSAT tumour stage at diagnosis, n(%)**	
1	86 (9)
2	474 (50)
3	218 (23)
4	164 (17)
**mENSAT tumour stage at diagnosis, n(%)***	
4a	84 (9)
4b	35 (4)
4c	14 (1)
Unknown	31 (3)
**Ki67 index**	18 (8, 30)
**Ki67 index (categories), n(%)**	
0-9	264 (28)
10-19	216 (23)
≥20	462 (49)

**Resection status of primary tumour, n(%)**	
R0	648 (69)
RX	104 (11)
R1	56 (6)
R2	134 (14)
**S-GRAS group, n(%)**	
0-1	168 (18)
2-3	366 (39)
4-5	225 (24)
6-9	183 (19)
**Disease recurrence, progress or death^, n(%)**	572 (61)
**Disease-specific death, n(%)**	281 (30)
**Death of any cause, n(%)**	295 (31)
**Median progression-free survival, months (95% CI)**	21 (19, 24)
**Median disease-specific survival, months (95% CI)**	123 (89, not reached)
**Median overall survival, months (95% CI)**	104 (84, 164)

Continuous variables presented as median (and interquartile range).

*modified ENSAT staging for patients with metastatic disease at diagnosis (8) ^disease-related death

Median follow-up times: PFS: 49.63 months (95% CI 46.03, 58.77) and DSS: 45.00 months (95% CI 41.00, 48.00).
