# Supplementary material for: S-GRAS score for prognostic classification of adrenocortical carcinoma: an international, multicenter ENSAT study
Source: Eur J Endocrinol. 2021 Oct 27;186(1):25–36. doi: 10.1530/EJE-21-0510 (PMC8679848; doi:10.1530/EJE-21-0510)
Supplement: Supplementary Table 5 – Multivariable Cox Regression model for progression-free survival (A) and disease-specific survival (B) with and without resection status, age and symptoms (RAS components) added to ENSAT tumour stage (S) and ki67 index (G). [file supplementary_table_5.pdf]

Supplementary Table 5 – Multivariable Cox Regression model for progression-free survival (A) and disease-specific survival (B) with and without resection status, age and symptoms (RAS components) added to ENSAT tumour stage (S) and ki67 index (G).

**A) Multivariable cox regression model (PFS)**				
	**ENSAT stage and ki67 only**		**All five variables in S-GRAS score**	
**Variables**	**Hazard ratio (95% CI)**	**p-value**	**Hazard ratio (95% CI)**	**p-value**
**ENSAT stage**				
1	1		1	
2	1.75 (1.17, 2.61)	0.006	1.55 (1.04, 2.32)	0.033
3	3.09 (2.04, 4.67)	<0.0001	2.64 (1.74, 4.01)	<0.0001
4	5.23 (3.43, 7.98)	<0.0001	2.77 (1.62, 4.76)	<0.0001
**Ki67 index**				
0-9	1		1	
10-19	2.08 (1.58, 2.73)	<0.0001	1.99 (1.51, 2.62)	<0.0001
≥20	3.25 (2.55, 4.14)	<0.0001	2.86 (2.24, 3.66)	<0.0001
**Resection status**				
R0			1	
RX	-	-	1.55 (1.21, 2.00)	0.001
R1	-	-	1.66 (1.19, 2.31)	0.003
R2	-	-	2.03 (1.33, 3.09)	0.001
**Age**				
<50 years			1	
≥50 years	-	-	1.16 (0.98, 1.37)	0.083
**Symptoms**				
No			1	
Yes	-	-	1.55 (1.26, 1.90)	<0.0001

**B) Multivariable cox regression model (DSS)**				
	**ENSAT stage and ki67 only**		**All five variables**	
**Variables**	**Hazard ratio (95% CI)**	**p-value**	**Hazard ratio (95% CI)**	**p-value**
**ENSAT stage**				
1	1		1	
2	2.03 (0.99, 4.19)	0.055	1.77 (0.86, 3.66)	0.124
3	4.01 (1.93, 8.33)	<0.0001	3.44 (1.64, 7.19)	0.001

4	10.32 (4.96, 21.50)	<0.0001	4.17 (1.77, 9.84)	0.001
**Ki67 index**				
0-9	1		1	
10-19	2.00 (1.27, 3.16)	0.003	2.01 (1.27, 3.17)	0.003
≥20	4.03 (2.73, 5.94)	<0.0001	3.55 (2.38, 5.27)	<0.0001
**Resection status**				
R0			1	
RX	-	-	1.31 (0.88, 1.95)	0.185
R1	-	-	1.65 (1.05, 2.60)	0.030
R2	-	-	2.79 (1.63, 4.77)	<0.0001
**Age**				
<50 years			1	
≥50 years	-	-	1.50 (1.18, 1.91)	0.001
**Symptoms**				
No			1	
Yes	-	-	2.20 (1.59, 3.05)	<0.0001
