# Supplementary material for: S-GRAS score for prognostic classification of adrenocortical carcinoma: an international, multicenter ENSAT study
Source: Eur J Endocrinol. 2021 Oct 27;186(1):25–36. doi: 10.1530/EJE-21-0510 (PMC8679848; doi:10.1530/EJE-21-0510)
Supplement: Suppl Table 6. Univariate survival analysis: median disease-specific survival (DSS), percentage survival at 24 and 60 months, hazard ratios and discrimination analysis of single prognostic variables and S-GRAS scoring. [file supplementary_table_6.pdf]

Suppl Table 6. Univariate survival analysis: median disease-specific survival (DSS), percentage survival at 24 and 60 months, hazard ratios and discrimination analysis of single prognostic variables and S-GRAS scoring.

Variables	N	% survival at 24 months	% survival at 60 months	Median DSS, months (95% CI)	Hazard ratio (95% CI)	p-value (HR)	Harrell's C index (95% CI)	p-values (Harrell's C)	Royston-Sauerbrei's R^2_D statistic
**ENSAT stage**									
1	86	97.4 (89.9, 99.3)	87.8 (75.5, 94.2)	NR	1	Reference			
2	474	90.2 (86.8, 92.8)	72.9 (67.2, 77.8)	248.8 (131.2, NR)	2.6 (1.3, 5.3)	0.010	0.72 (0.69, 0.75)	<0.0001* <0.0001**	0.35 (0.28, 0.42)
3	218	75.3 (68.1, 81.1)	53.6 (44.6, 61.8)	66.0 (53.0, 123.0)	5.6 (2.7, 11.6)	<0.0001			
4	164	45.5 (36.6, 53.9)	21.3 (12.9, 31.1)	19.0 (16.5, 30.0)	16.8 (8.1, 34.7)	<0.0001			
**Ki67 index**									
0-9	264	97.2 (93.8, 98.7)	87.3 (81.0, 91.7)	248.8 (131.2, NR)	1	Reference	0.69 (0.66, 0.71)	<0.0001* <0.0001**	0.31 (0.22, 0.39)
10-19	216	85.6 (79.3, 90.0)	72.4 (63.8, 79.3)	163.9 (123.0, NR)	2.2 (1.4, 3.5)	0.001			
≥20	462	68.0 (63.0, 72.4)	41.7 (35.6, 47.7)	49.6 (36.5, 57.0)	5.6 (3.8, 8.2)	<0.0001			
Ki67 (continuous)	*942*	-	-	-	*1.03 (1.02, 1.04)*	*<0.0001*	*0.71 (0.68, 0.74)*	*<0.0001*, <0.0001***	*0.21 (0.15, 0.27)*
**Resection status**									
R0	648	89.5 (86.5, 91.8)	73.1 (68.3, 77.2)	248.8 (131.2, NR)	1	Reference			
RX	104	77.1 (66.1, 84.9)	63.8 (51.2, 73.9)	72.8 (63.8, NR)	1.9 (1.3, 2.8)	0.002	0.70 (0.67, 0.73)	<0.0001* <0.0001**	0.36 (0.29, 0.42)
R1	56	69.5 (53.9, 80.7)	25.7 (9.9, 44.9)	44.5 (28.4, 60.0)	3.2 (2.1, 4.9)	<0.0001			
R2	134	38.8 (29.2, 48.2)	13.0 (5.8, 23.3)	17.0 (14.6, 23.0)	8.1 (6.1, 10.8)	<0.0001			
**Age**									
<50 years	466	82.1 (77.8, 85.5)	64.6 (58.8, 69.8)	147.5 (99.0, NR)	1	Reference	0.53 (0.50, 0.56)	<0.0001* <0.0001**	0.02 (0.00, 0.05)
≥50 years	476	78.3 (73.9, 82.1)	59.0 (52.9, 64.7)	89.0 (66.0, NR)	1.3 (1.0, 1.6)	0.034			
Age (continuous)	*942*	-	-	-	*1.01 (1.00, 1.02)*	*0.033*	*0.54 (0.50, 0.58)*	*<0.0001*, <0.0001***	*0.01 (0.00, 0.04)*
**Symptoms**									
No	277	93.1 (88.7, 95.8)	78.1 (70.5, 83.9)	248.8 (147.5, NR)	1	Reference	0.60 (0.58, 0.62)	<0.0001* <0.0001**	0.16 (0.07, 0.25)
Yes	665	75.1 (71.2, 78.4)	55.4 (50.4, 60.1)	84.0 (63.0, 123.0)	2.5 (1.8, 3.5)	<0.0001			
**S-GRAS group**									
0-1	168	99.2 (94.3, 99.9)	93.5 (86.8, 96.9)	248.8 (147.5, NR)	1	Reference			
2-3	366	91.8 (88.0, 94.4)	72.0 (65.3, 77.7)	163.9 (123.0, NR)	3.4 (1.9, 6.3)	<0.0001	0.77 (0.74, 0.79)	Reference 1*	0.46 (0.39, 0.52)
4-5	225	76.1 (69.2, 81.7)	56.1 (47.1, 64.2)	73.0 (55.8, NR)	7.8 (4.2, 14.5)	<0.0001			
6-9	183	41.9 (33.6, 50.0)	12.8 (6.4, 21.6)	20.0 (16.6, 25.6)	27.3 (14.9, 50.0)	<0.0001			
**S-GRAS individual score**									
0	36	100	95.2 (70.7, 99.3)	NR	0.3 (0.0, 2.5)	0.285			
1	132	98.9 (92.7, 99.9)	93.0 (85.0, 96.8)	248.8 (147.5, NR)	1	Reference			
2	155	93.5 (87.3, 96.7)	80.2 (70.1, 87.2)	163.9 (131.2, NR)	2.1 (1.0, 4.2)	0.045			
3	211	90.5 (85.2, 94.0)	66.0 (56.6, 73.8)	123.0 (71.0, NR)	3.6 (1.9, 6.9)	<0.0001			
4	153	79.9 (71.7, 85.9)	60.4 (49.5, 69.6)	73.0 (56.5, NR)	5.7 (2.9, 11.0)	<0.0001	0.79 (0.76, 0.82)	Reference 2**	0.45 (0.39, 0.50)
5	72	68.1 (54.2, 78.5)	44.6 (27.9, 60.1)	33.0 (29.0, NR)	10.00 (4.9, 20.3)	<0.0001			
6	32	66.4 (43.6, 81.7)	24.1 (6.9, 46.9)	35.0 (21.0, 52.4)	13.4 (6.2, 28.9)	<0.0001			
7	39	42.0 (23.7, 59.2)	33.6 (14.6, 53.9)	22.6 (15.0, NR)	20.3 (9.6, 42.9)	<0.0001			
8	75	37.6 (25.3, 49.9)	9.3 (2.8, 20.7)	16.6 (13.2, 24.0)	27.1 (14.0, 52.6)	<0.0001			

9	37	30.8 (14.7, 48.4)	-	15.9 (8.5, 23.0)	39.5 (19.2, 81.5)	<0.0001			

NR, not reached (= median survival not reached i.e. percentage survival remained >50% so this value and/or its 95% confidence intervals cannot be computed). *p-values comparing the variable with the S-GRAS group. **p-values comparing the variable with the S-GRAS scores.
