# Supplementary material for: S-GRAS score for prognostic classification of adrenocortical carcinoma: an international, multicenter ENSAT study
Source: Eur J Endocrinol. 2021 Oct 27;186(1):25–36. doi: 10.1530/EJE-21-0510 (PMC8679848; doi:10.1530/EJE-21-0510)
Supplement: Suppl Table 7. Demographic data for patients with adrenocortical carcinoma in the adjuvant mitotane cohort (n=795) [file supplementary_table_7.pdf]

Suppl Table 7. Demographic data for patients with adrenocortical carcinoma in the adjuvant mitotane cohort (n=795)

Variable	No mitotane adjuvant	Mitotane adjuvant	P value
**N**	314	481	-
**Age, years**	51 (39, 61)	49 (38, 60)	*0.0824*
**Male, n(%)**	131 (41.7)	175 (36.4)	*0.131*
**Symptoms at diagnosis, n(%)**	198 (63.1)	338 (70.3)	***0.034***
**Symptom type, n(%)**			*0.257*
None	116 (36.9)	143 (29.7)	
Tumour mass	57 (18.2)	88 (18.3)	
Steroids	114 (36.3)	198 (41.2)	
Cancer-related	10 (3.2)	22 (4.6)	
Combinations of the above	17 (5.4)	30 (6.2)	
**Hormone pattern, n(%)**			*0.739*
Non-functional	131 (41.7)	192 (39.9)	
Glucocorticoid excess	59 (18.8)	101 (21.0)	
Androgen excess	26 (8.3)	40 (8.3)	
Combinations of the above or other	69 (21.9)	114 (23.7)	
Unknown	29 (9.2)	34 (7.1)	
**ENSAT tumour stage at diagnosis, n(%)**			***0.017***
1	47 (15.0)	38 (8.0)	
2	172 (54.8)	291 (60.5)	
3	80 (25.5)	130 (27.0)	
4	15 (4.8)	22 (4.6)	
**Ki67 index**	10 (5, 20)	18 (10, 30)	**< *0.0001***
**Ki67 index (categories), n(%)**			**< *0.0001***
0-9	141 (44.9)	112 (23.3)	
10-19	68 (21.7)	130 (27.0)	
≥20	105 (33.4)	239 (49.7)	
**Resection status of primary tumour, n(%)**			*0.357*
R0	260 (82.8)	379 (78.8)	
RX	36 (11.5)	65 (13.5)	
R1	18 (5.73)	37 (7.7)	
**S-GRAS group, n(%)**			**< *0.0001***
0-1	98 (31.21)	68 (14.1)	
2-3	122 (38.9)	238 (49.5)	
4-5	76 (24.2)	144 (29.94)	
6-9	18 (5.7)	31 (6.4)	
**Disease recurrence, progress or death*, n(%)**	176 (56.1)	267 (55.5)	*0.881*
**Disease-specific death, n(%)**	80 (25.5)	112 (23.3)	*0.480*
**Death of any cause, n(%)**	83 (26.4)	121 (25.2)	*0.687*

Continuous variables presented as median (and interquartile range).*disease-related death.
