# Supplementary material for: S-GRAS score for prognostic classification of adrenocortical carcinoma: an international, multicenter ENSAT study
Source: Eur J Endocrinol. 2021 Oct 27;186(1):25–36. doi: 10.1530/EJE-21-0510 (PMC8679848; doi:10.1530/EJE-21-0510)
Supplement: Suppl Table 8. Univariate survival analysis: median disease-specific survival (DSS), hazard ratios and discrimination analysis of single prognostic variables and S-GRAS scoring in patients with adrenocortical carcinoma treated or not treated with mitotane adjuvant (n=795). [file supplementary_table_8.pdf]

Suppl Table 8. Univariate survival analysis: median disease-specific survival (DSS), hazard ratios and discrimination analysis of single prognostic variables and S-GRAS scoring in patients with adrenocortical carcinoma treated or not treated with mitotane adjuvant (n=795).

Variables	Mitotane treatment	N	Median DSS, months (95% CI)	Hazard ratio (95% CI)	p-value (HR)	HR difference - untreated vs treated (95% CI)	p-value (difference)	Harrell's C index (95% CI)	Royston-Sauerbrei's R^2_D statistic
**ENSAT stage**									
1	No	47	NR (97.4, NR)	1	Reference			0.69 (0.64, 0.75)	0.24 (0.12, 0.37)
2		172	NR (99.0, NR)	3.2 (1.1, 9.0)	0.027				
3		80	54.57 (34.0, 71.0)	8.9 (3.2, 25.2)	<0.0001				
4		15	NR (14.6, NR)	10.8 (2.9, 40.4)	<0.0001				
1	Yes	38	NR	1.7 (0.4, 6.9)	0.444	0.72 (-1.66, 3.10)	0.555	0.61 (0.56, 0.66)	0.14 (0.06, 0.25)
2		291	147.5 (127.0, NR)	3.2 (1.2, 8.8)	0.026	-0.04 (-1.37, 1.30)	0.958		
3		130	123.0 (56.5, NR)	5.5 (2.0, 15.4)	0.001	-3.44 (-8.26, 1.38)	0.162		
4		22	46.0 (18.0, 84.0)	13.8 (4.5, 42.4)	<0.0001	2.99 (-9.45, 15.44)	0.637		
**Ki67 index**									
0-9	No	141	NR (99.0, NR)	1	Reference			0.72 (0.67, 0.77)	0.30 (0.16, 0.43)
10-19		68	163.9 (54.6, NR)	2.7 (1.4, 5.3)	0.004				
≥20		105	48.0 (34.0, 73.0)	5.3 (3.0, 9.4)	<0.0001				
0-9	Yes	112	248.8 (131.2, NR)	0.9 (0.4, 1.9)	0.811	-0.09 (-0.77, 0.60)	0.802	0.65 (0.61, 0.69)	0.25 (0.12, 0.38)
10-19		130	147.5 (123.0, NR)	1.8 (1.0, 3.5)	0.063	-0.85 (-2.35, 0.65)	0.267		
≥20		239	69.0 (56.5, NR)	3.9 (2.3, 6.7)	<0.0001	-1.38 (-3.29, 0.54)	0.158		
**Resection status**									
R0	No	260	NR (163.9, NR)	1	Reference			0.61 (0.55, 0.66)	0.20 (0.09, 0.33)
RX		36	63.8 (30.0, 83.1)	2.8 (1.6, 5.0)	0.001				
R1		18	41.0 (14.6, 50.0)	4.2 (2.1, 8.2)	<0.0001				
R0	Yes	379	147.5 (123.0, NR)	1.1 (0.8, 1.6)	0.585	0.10 (-0.28, 0.48)	0.602	0.56 (0.51, 0.60)	0.08 (0.01, 0.19)
RX		65	73.0 (68.0, NR)	1.5 (0.9, 2.7)	0.151	-1.27 (-2.94, 0.40)	0.136		
R1		37	52.4 (28.4, NR)	2.9 (1.6, 5.2)	<0.0001	-1.26 (-4.28, 1.75)	0.412		
**S-GRAS group**									
0-1	No	98	NR	1	Reference			0.76 (0.71, 0.81)	0.42 (0.30, 0.53)
2-3		122	163.9 (71.0, NR)	3.6 (1.6, 8.3)	0.002				
4-5		76	41.0 (28.6, NR)	9.6 (4.2, 21.9)	<0.0001				
6-9		18	18.1 (11.0, 35.0)	34.0 (13.5, 86.0)	<0.0001				
0-1	Yes	68	248.8 (147.5, NR)	1.0 (0.3, 3.0)	0.923	-0.05 (-1.14, 1.03)	0.921	0.68 (0.64, 0.73)	0.27 (0.16, 0.37)
2-3		238	NR (123.0, NR)	3.2 (1.4, 7.1)	0.004	-0.45 (-2.09, 1.19)	0.592		
4-5		144	73.0 (55.8, NR)	7.1 (3.2, 15.8)	<0.0001	-2.44 (-6.89, 2.00)	0.281		
6-9		31	30.4 (21.0, 63.0)	15.5 (6.4, 38.0)	<0.0001	-18.49 (-42.89, 5.91)	0.137		

HR, hazard ratio; NR, not reached (= median survival not reached i.e. percentage survival remained >50% so this value and/or its 95% confidence intervals cannot be computed).
