# Supplementary material for: S-GRAS score for prognostic classification of adrenocortical carcinoma: an international, multicenter ENSAT study
Source: Eur J Endocrinol. 2021 Oct 27;186(1):25–36. doi: 10.1530/EJE-21-0510 (PMC8679848; doi:10.1530/EJE-21-0510)
Supplement: Suppl Table 9. Overview of different available models for prognostication in patients with ACC compared to the present series. [file supplementary_table_9.pdf]

Suppl Table 9. Overview of different available models for prognostication in patients with ACC compared to the present series.

Study	N	Outcome	Predictors	Harrell's C-index
Kim 2016	148	RFS and OS (curative surgery)	Tumour size, nodal status, T stage, cortisol secretion, capsular invasion, resection status	RFS 0.74<br>OS 0.70
Li 2018	751	DSS and OS	Age, year of diagnosis, histology grade, tumour stage, chemotherapy	DSS 0.67<br>OS 0.68
Kong 2019	404	OS (surgery)	Age, T stage, N stage, M stage	OS 0.72
Ettaieb 2020	160	DSS	Age, modified ENSAT stage, resection status (+/- ki67 index)	DSS 0.77
Baechle 2021	243	OS and RFS	Age, symptoms (hypersecretion), grade (Weiss score or ki67), resection status	Not Available
**Present study**	**942**	**PFS and DSS (surgery)**	**Age, symptoms, ENSAT stage, ki67 index, resection status (S-GRAS score)**	**PFS 0.71/0.73***<br>**DSS 0.77/0.79**

RFS=recurrence-free survival, OS=overall survival, DSS= disease-specific survival, PFS=progression-free survival, *S-GRAS as groups and as continuous variable
