# Supplementary material for: S-GRAS score for prognostic classification of adrenocortical carcinoma: an international, multicenter ENSAT study
Source: Eur J Endocrinol. 2021 Oct 27;186(1):25–36. doi: 10.1530/EJE-21-0510 (PMC8679848; doi:10.1530/EJE-21-0510)
Supplement: Supplementary Table 10. Overview of our hypothetically proposed “S-GRAS-based recommendation” as compared with the current European ACC Guidelines (Fassnacht et al EJE 2018). [file supplementary_table_10.pdf]

Supplementary Table 10. Overview of our hypothetically proposed "*S-GRAS-based recommendation*" as compared with the current European ACC Guidelines (Fassnacht et al EJE 2018).

	N (% of total)	SGRAS 0-1 (n)	S GRAS 2-3 (n)	S-GRAS 4-5 (n)	S-GRAS 6-9 (n)
S-GRAS-based "recommendation"→		*No mitotane*	*No mitotane*	*Adjuvant mitotane*	*Adjuvant EDP-M/EP-M*
Recommendation in ACC guidelines ↓					
R0 and ENSAT 1-2 and ki67 ≤10%<br>Consider mitotane or surveillance	242 (26%)	**157**	**85**	**0**	**0**
R0 and ENSAT 3-4 OR<br><br>ENSAT 1-2 AND ki67 > 10%<br>Adjuvant mitotane	177 (19%)<br><br>345 (37%)	1<br><br>7	81<br><br>196	**92**<br><br>**139**	3<br><br>3
R0 and ENSAT 3-4 AND<br>ENSAT 1-2 AND ki67 > 10%<br>Adjuvant mitotane	116 (12%)	0	31	**82**	3
RX / R1<br>Adjuvant mitotane + consider radiotherapy (ev. chemotherapy in higher risk patients)	160 (17%)	3	35	**76**	**180**

Legend:
Green shading boxes = "our suggestion" is a less aggressive approach (surveillance vs adjuvant mitotane).
Light grey shading boxes and bold text = agreement between guidelines and "our suggestion" for adjuvant mitotane.
Light red shading = "our suggestion" favours a more aggressive adjuvant treatment (adjuvant chemotherapy).

This table shows that in the large majority of the cases, our proposed "*S-GRAS-based recommendations*" are in agreement with the current guidelines. However, there are subgroups of patients for which, according to the S-GRAS stratification, we would theoretically not suggest adjuvant mitotane.
